# Folate as a potential treatment for lethal ventricular arrhythmias in TANGO2-deficiency disorder

**DOI:** 10.1172/jci.insight.171005

**Published:** 2024-06-10

**Authors:** Weiyi Xu, Yingqiong Cao, Sara B. Stephens, Maria Jose Arredondo, Yifan Chen, William Perez, Liang Sun, Andy C. Yu, Jean J. Kim, Seema R. Lalani, Na Li, Frank T. Horrigan, Francisco Altamirano, Xander H.T. Wehrens, Christina Y. Miyake, Lilei Zhang

**Affiliations:** 1 Department of Molecular and Human Genetics, Baylor College of Medicine, Houston, Texas, USA.; 2 Department of Pediatrics, Division of Pediatric Cardiology, Texas Children’s Hospital and Baylor College of Medicine, Houston, Texas, USA.; 3 Department of Cardiovascular Sciences, Houston Methodist Research Institute, Houston, Texas, USA.; 4 Department of Integrative Physiology,; 5 Department of Molecular and Cellular Biology,; 6 Human Stem Cell Core, Advanced Technology Cores,; 7 Department of Medicine (Section of Cardiovascular Research), and; 8 Cardiovascular Research Institute, Baylor College of Medicine, Houston, Texas, USA.; 9 Department of Cardiothoracic Surgery, Weill Cornell Medical College, Cornell University, Ithaca, New York, USA.; 10 Department of Neuroscience,; 11 Department of Pediatrics,; 12 Center for Space Medicine, and; 13 Department of Molecular Physiology and Biophysics, Baylor College of Medicine, Houston, Texas, USA.

**Keywords:** Cardiology, Genetics, Arrhythmias, iPS cells

## Abstract

TANGO2-deficiency disorder (TDD) is an autosomal-recessive genetic disease caused by biallelic loss-of-function variants in the *TANGO2* gene. TDD-associated cardiac arrhythmias are recalcitrant to standard antiarrhythmic medications and constitute the leading cause of death. Disease modeling for TDD has been primarily carried out using human dermal fibroblast and, more recently, in *Drosophila* by multiple research groups. No human cardiomyocyte system has been reported, which greatly hinders the investigation and understanding of TDD-associated arrhythmias. Here, we established potentially novel patient-derived induced pluripotent stem cell differentiated cardiomyocyte (iPSC-CM) models that recapitulate key electrophysiological abnormalities in TDD. These electrophysiological abnormalities were rescued in iPSC-CMs with either adenoviral expression of WT-TANGO2 or correction of the pathogenic variant using CRISPR editing. Our natural history study in patients with TDD suggests that the intake of multivitamin/B complex greatly diminished the risk of cardiac crises in patients with TDD. In agreement with the clinical findings, we demonstrated that high-dose folate (vitamin B9) virtually abolishes arrhythmias in TDD iPSC-CMs and that folate’s effect was blocked by the dihydrofolate reductase inhibitor methotrexate, supporting the need for intracellular folate to mediate antiarrhythmic effects. In summary, data from TDD iPSC-CM models together with clinical observations support the use of B vitamins to mitigate cardiac crises in patients with TDD, providing potentially life-saving treatment strategies during life-threatening events.

## Abstract 2

Combined analysis of patient-derived iPSC-cardiomyocytes and clinical observations support the use of B vitamins to mitigate cardiac crises in patients with TANGO2 deficiency disorder.

## Graphical abstract

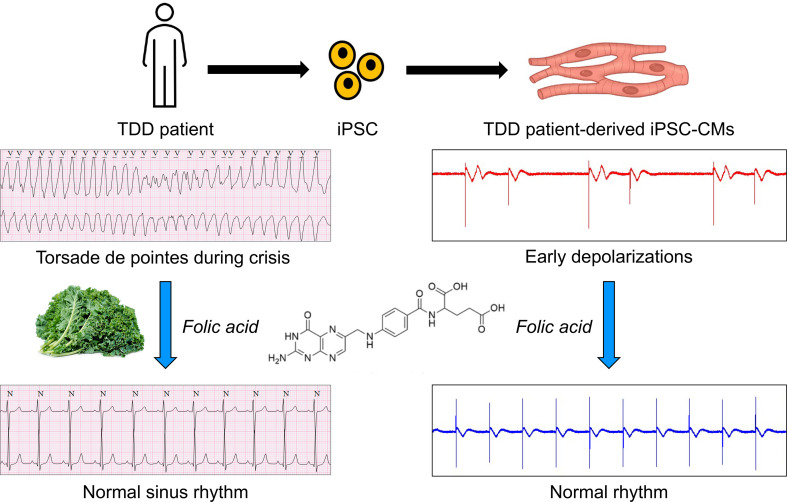

## Introduction

Biallelic loss-of-function variants in *TANGO2* cause TANGO2-deficiency disorder (TDD, OMIM #616878), a rare multiorgan disorder associated with significant morbidity that is estimated to affect ~8,000 individuals worldwide (1–15). While chronic symptoms are predominantly neurodevelopmental, patients are susceptible to stressors such as febrile illnesses or fasting, which can trigger metabolic crises. Metabolic crises are acute episodes of worsening clinical symptoms associated with ataxia, muscle weakness, hypoglycemia, rhabdomyolysis, and death.

During a metabolic crisis, 65% of patients will develop acute arrhythmias or cardiac systolic dysfunction, and these more lethal events are termed cardiac crises (15). Despite normal cardiac structure and function at baseline, ventricular arrhythmias during cardiac crises are the leading cause of death in TDD because these arrhythmias usually do not respond to standard antiarrhythmic therapies, including β-adrenergic blockers, lidocaine, flecainide, amiodarone, potassium, calcium, and sympathetic denervation (1, 16). In a recent study of 27 patients with cardiac crisis, the mortality rate was 37% (3). During TDD-related crises, QT (interval between the start of Q wave and the end of T wave) prolongation, often marked, occurs in all patients (15, 16). Patients also develop frequent isolated premature ventricular contractions (PVCs) that often herald hemodynamically significant episodes of Torsade de Pointes (TdP) or polymorphic ventricular tachycardia.

Since the molecular mechanisms underlying TDD remain largely unclear with conflicting proposed hypotheses (2, 5, 13, 14, 17), effective treatment strategies for TDD are lacking and mortality from cardiac arrhythmias remains high (16). Therefore, a model that recapitulates key cardiac electrophysiological abnormalities in TDD is needed to investigate disease mechanism and identify novel therapeutic strategies to ameliorate the high mortality rates. Here, we developed patient-derived induced pluripotent stem cell differentiated cardiomyocyte (iPSC-CM) models from 2 patients with TDD with different causative variants and genetic backgrounds. We observed electrical abnormalities in these cells that recapitulate key findings seen in patients’ electrocardiograms (ECGs). These abnormalities can be eliminated by either reexpressing WT TANGO2 or CRISPR correction of the specific mutation, supporting the validity of these models. Based on findings from 2 independent isogenic iPSC-CM models and a clinical natural history study, we made a striking observation that B vitamins — in particular, folate (vitamin B9) — can prevent or suppress TDD associated arrhythmias, and that may have profound implication on the acute treatment of this currently lethal arrhythmic disease.

## Results

### Patient-derived iPSC-CM model recapitulates key arrhythmic phenotypes in TDD.

**Figure 1:**
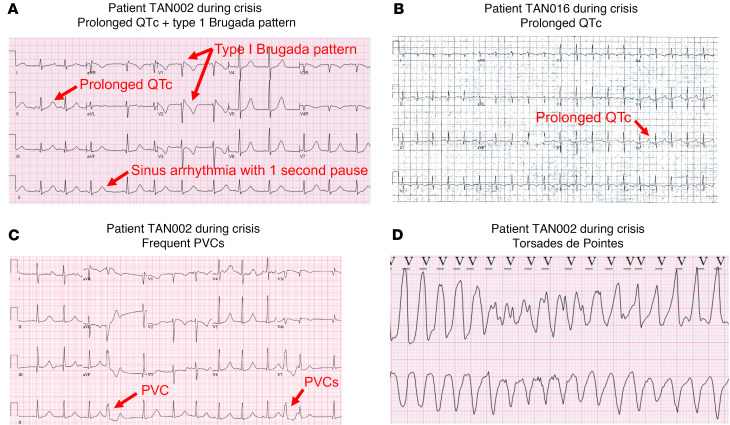
Electrocardiographic (ECG) abnormalities in patient TAN002 and TAN016 during metabolic crises. (**A**) Prolonged QTc and intermittent type I Brugada pattern in patient TAN002. (**B**) ECG showing prolonged QTc in TAN016 patient during crisis. (**C**) Premature ventricular contractions (PVCs) in patient TAN002 during metabolic crisis. (**D**) ECG showing torsades de pointes (TdP) in patient TAN002 during metabolic crises. Scale bar: 25 mm/sec for *x* axis and 100 mm/mV for *y* axis.

**Figure 2:**
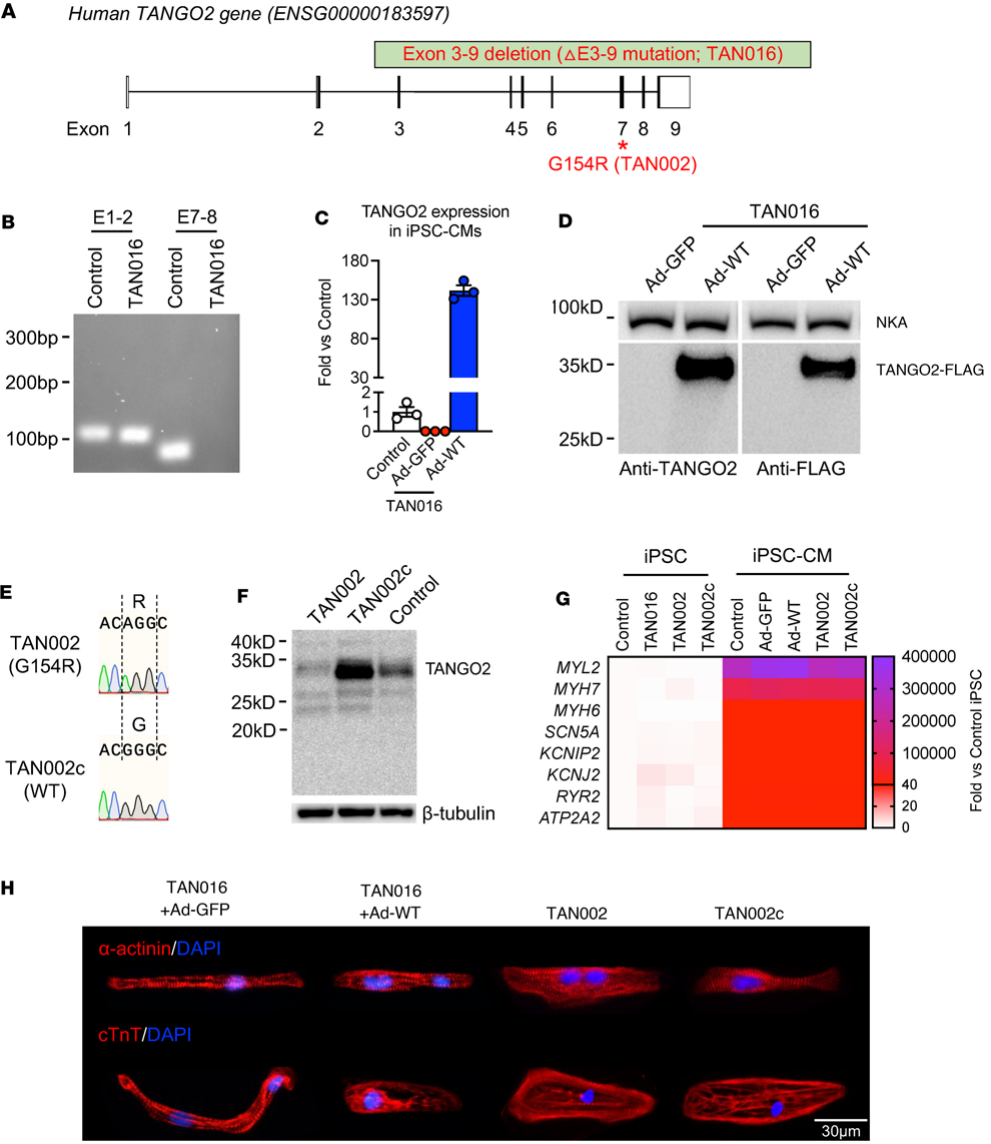
Generation of patient-derived iPSC-CMs and isogenic controls from TAN016 and TAN002. (**A**) G154R and ΔE3–9 variants in human *TANGO2* locus. Coding regions in exons are shown in black. (**B**) RT-PCR for TAN016 using primer pairs targeting exon 1–2 (E1–2) or exon 7–8 (E7–8) of WT-TANGO2 transcript. Sample from an unrelated WT line was used as positive control. (**C**) Transcriptional level of TANGO2 in the control, TAN016+Ad-GFP (Ad-GFP), and TAN016+Ad-WT (Ad-WT) iPSC-CMs. Quantification was performed by RT-PCR and normalized to the level of the control iPSC-CMs (*n* = 3). *PPIB* was used as internal control. Data are mean ± SEM. (**D**) Immunoblotting showing the adenoviral expression of WT-TANGO2 protein in TAN016 iPSC-CMs. A 3XFLAG tag was added to the C-terminus of the ectopic TANGO2 protein. Image on the right demonstrates the specificity of TANGO2 polyclonal antibody using an anti-FLAG antibody. Na^+^/K^+^ ATPase (NKA) was used as loading control. (**E**) Sanger sequencing confirmed the genotypes in TAN002 and its isogenic correction line TAN002c. (**F**) Immunoblotting showing the TANGO2 protein level in TAN002, TAN002c, and control iPSC-CMs detected by the TANGO2 polyclonal antibody. β-Tubulin was used as loading control. (**G**) Transcriptional level of key cardiac marker genes in 4 iPSC and 5 iPSC-CM lines used in the study. Quantification was performed by RT-PCR and normalized to control iPSC group (*n* = 3–6). *PPIB* was used as internal control. Mean value for each line is presented in the heatmap. (**H**) Immunofluorescence staining of α-actinin (top) and cardiac troponin T (cTnT; bottom) in TAN016+Ad-GFP/WT-TANGO2, TAN002, and TAN002c. Nuclei were stained by DAPI in blue. Scale bar: 30 μm.

We generated 2 independent patient-derived iPSC-CM lines from 2 patients with TDD, TAN016 and TAN002. Both patients developed prolonged QTc (heart rate-corrected QT) during metabolic crises (Figure 1, A and B). TAN002 also demonstrated a type I Brugada-ECG pattern, short pauses, and frequent PVCs prior to the onset of lethal ventricular arrhythmias (16) (Figure 1, A and C). TdP was observed from inpatient telemetry monitoring of TAN002 during crisis (Figure 1D). Genetic testing showed TAN016 harbors homozygous exons 3–9 deletion (ΔE3–9), which is the most common genetic variant seen in patients with TDD (15) and is recurring in patients with an European ancestry (Figure 2A). TAN002 harbors the homozygous c.460G > A (p.G154R) variant, the second most common genetic variant and a recurring variant among patients with a Hispanic/Latino ancestry (3).

To eliminate variation introduced by genetic background between different cell lines, we generated corresponding isogenic control lines for TAN016 and TAN002 iPSC-CMs. In TAN016, ΔE3–9 leads to an in-frame deletion resulting in a truncated transcript of exon 1–2 only (Figure 2B), which interestingly does not undergo significant nonsense mediated decay. However, exon 1–2 only encodes for the first 9 amino acids, which are part of the mitochondria targeting signal peptide, thus the truncated transcript is unlikely to result in a stable or functional TANGO2 protein. In fact, we found that no TANGO2 protein was produced using a polyclonal antibody that detects the full-length TANGO2 (i.e., first lane on the left in Figure 2D). Thus, we consider the TAN016 iPSC-CMs as a de facto TANGO2-KO line. We then generated a set of 2 isogenic iPSC-CMs on the TAN016 background by expressing WT-TANGO2 or GFP control using an adenoviral vector (TAN016+Ad-WT or TAN016+Ad-GFP). The adenovirus was titrated and applied at the lowest concentration, allowing 100% infection efficiency in iPSC-CMs (Supplemental Figure 1; supplemental material available online with this article; undefinedDS1). Using quantified PCR (qPCR), we first confirmed the *TANGO2* expression in TANG016+Ad-WT at transcripts level, while no transcription of full-length *TANGO2* was detected in TAN016+Ad-GFP (Figure 2C). Due to the existence of the N-terminus signal peptide for mitochondria targeting, a 3XFLAG tag was added to the C-terminus to facilitate protein detection as previously reported (14). WT-TANGO2 expression in TAN016+Ad-WT was confirmed by both anti-TANGO2 antibody (13) and anti-FLAG antibody (Figure 2D). For TAN002 (G154R), an isogenic correction line denoted as TAN002c, was generated by CRISPR/Cas9-meditated genome editing (Figure 2E). Compared with the WT control, the G154R mutation results in a significantly lower TANGO2 protein abundance in TAN002 iPSC-CMs, which was rescued in the TAN002c iPSC-CMs (Figure 2F). All 4 iPSC-CM lines exhibited spontaneous contraction (Supplemental Videos 1–7) and high expression level of cardiac marker genes (18), which were demonstrated by both RT-PCR (Figure 2G and Supplemental Figure 2) and immunofluorescence staining (Figure 2H). After metabolic selection using glucose-free lactate medium (19), we were able to obtain a high purity cardiomyocyte population with over 99% cardiac troponin T^+^ (cTnT^+^) cells for all 4 iPSC-CM lines (Supplemental Figure 3). Moreover, we did not observe any significant difference in the expression level of the cardiac marker genes between the patient-derived iPSC-CM line and the corresponding isogenic line (TAN106+Ad-GFP versus TAN016+Ad-WT, or TAN002 versus TAN002c), suggesting that TANGO2 deficiency does not directly affect iPSC-CM differentiation or maturation. Therefore, these TDD iPSC-CM and their matching isogenic cell lines allow us to evaluate the effect of TANGO2 deficiency in human cardiomyocytes on multiple different isogenic genetic backgrounds.

**Figure 3:**
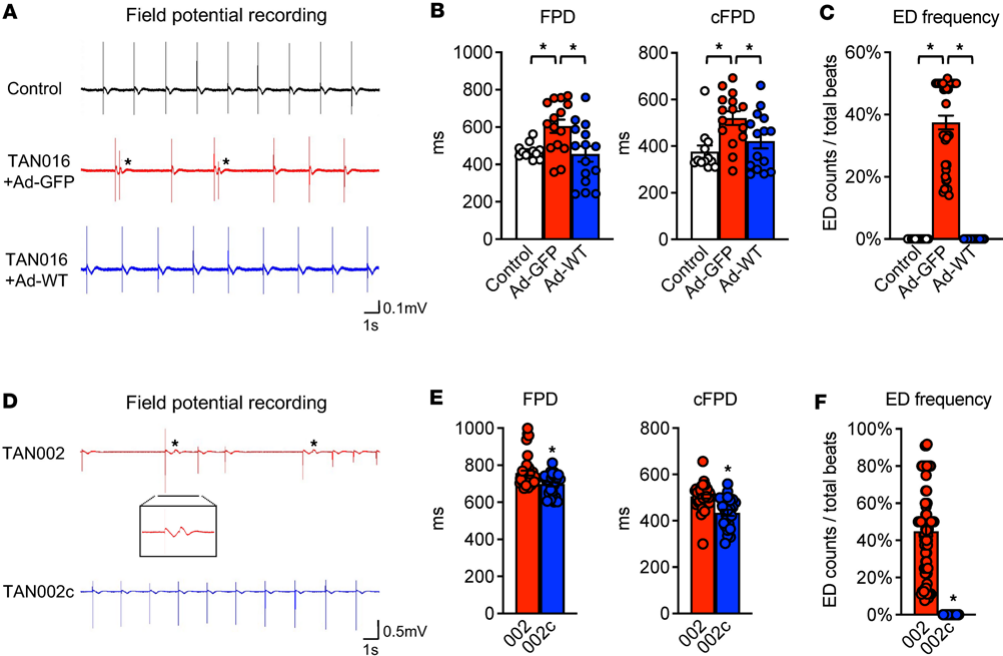
Patient-derived iPSC-CMs models recapitulate arrhythmias in TDD. (**A**) Representative field potential tracings of an independent control iPSC-CM line (BCMi002-A; top), TAN016 iPSC-CMs with adenoviral expression of GFP control (middle), or WT-TANGO2 (bottom). Asterisk indicates early depolarization (ED). (**B**) Quantifications of FPD (left panel) and cFPD (right panel) of control, TAN016+Ad-GFP, and TAN016+Ad-WT iPSC-CM lines (*n* = 12–15). (**C**) Quantification of early depolarization frequency of control, TAN016+Ad-GFP, and TAN016+Ad-WT iPSC-CM lines (*n* = 12–78). (**D**) Representative field potential tracings of TAN002 iPSC-CMs and its isogenic correction iPSC-CM line TAN002c. Asterisk indicates ED. Inset shows an ED in the enlarged tracing. (**E**) Quantifications of FPD (left panel) and cFPD (right panel) of TAN002 and TAN002c (*n* = 8–39). (**F**) Quantifications of ED frequency in TAN002 and TAN002c (*n* = 48–80). *P < 0.05, 1-way ANOVA using Dunnett’s correction with α = 0.05 for data in **B** and **C**, and unpaired 2-tailed Student *t* test for data in **E** and **F**. Data are mean ± SEM.

The arrhythmias in patients with TDD are episodic. The electrophysiology and cardiac function of these patients are entirely normal under baseline conditions (16). To investigate if TDD-associated arrhythmia can be recapitulated in TDD iPSC-CMs, we performed continuous real-time recording of the extracellular field potential (FP) using Agilent real-time cell analyzer (RTCA) CardioECR (20). In addition to the isogenic control, an independent control iPSC line (BCMi002-A) was also included for comparison. We found that TAN016 iPSC-CMs exhibited prolongation of FP duration (FPD), and FPD corrected by beating rate (cFPD) (Figure 3, A and B). The FPD measured from the noninvasive FP recording has been used as a surrogate for action potential duration or QT interval on ECG (20, 21). Therefore, the prolonged FPD/cFPD in the TAN016 iPSC-CM recapitulates the long QT phenotype that is seen in all patients with TDD during crisis (16). Moreover, in about 1% of the total recording time spanning several weeks, we observed episodic occurrences of early depolarizations (EDs), resembling PVCs, in TAN016+Ad-GFP iPSC-CMs. During these episodes with highly irregular beats (i.e., arrhythmia), EDs account for ~37.45% of the total beats (Figure 3, A and C). In contrast, the TAN016+Ad-WT showed normal rhythm without prolonged FPD/cFPD and EDs, confirming that the arrhythmias seen in the TAN016+Ad-GFP were indeed due to the loss of TANGO2 function. Consistently, TAN002 (G154R) displayed FPD and cFPD prolongation compared with TAN002c (Figure 3, D and E). In addition, episodes of frequent EDs were observed in TAN002 but never in TAN002c (Figure 3F). The ED frequency of TAN002 was ~44.9% during the arrhythmic episode, which is comparable with that of TAN016+Ad-GFP (Figure 3F). Collectively, these data show that our patient-derived iPSC-CM models faithfully recapitulated key arrhythmic phenotypes in TDD.

### Natural history study revealed a potential role of folate in preventing TDD-associated cardiac arrhythmias.

**Figure 4:**
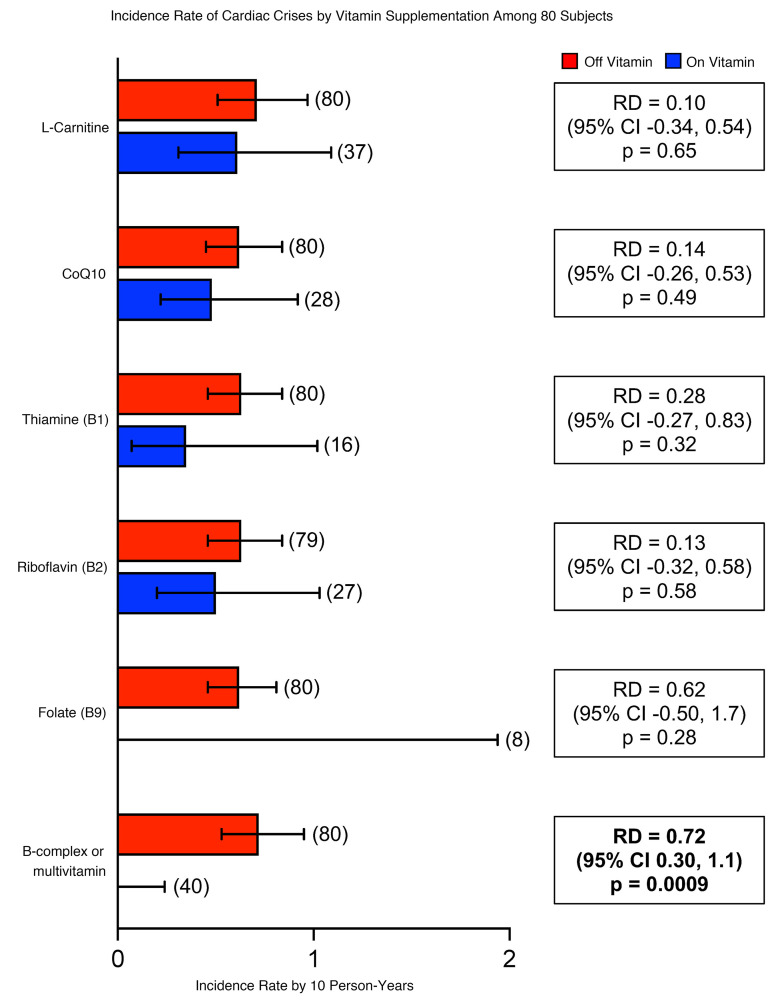
Clinical observations suggest folate may be an effective component in multivitamin/B complex to prevent TDD-associated arrhythmias. Incidence rate of cardiac crises by vitamin supplementation among 80 patients. Red bars indicate incident rate of cardiac crisis when patient was not taking the supplement. Blue bars indicated incident rate of cardiac crisis when patient was taking supplement. Patients taking folate and B complex or multivitamin had no cardiac crisis events. Incidence rates (IR) were computed by dividing the number of cardiac crises in each group by the sum of person-years in which subjects were taking the vitamin. Rate differences (RD) were computed by subtracting incidence rate among those taking the vitamin from the incidence rate among those not taking it (IR_off_ _vitamin_ – IR_on_ _vitamin_). The number of patients is indicated in the parenthesis above each error bar. RD, rate difference. Error bars show 95% CI, exact Poisson method.

As part of the largest natural history study for TDD with over 90 patients enrolled to date (ClinicalTrials.gov; accession no. NCT05374616), we have previously reported the negative association between B vitamin use and metabolic crisis (15). About 65% of patients with TDD developed life-threatening cardiac crisis during metabolic crisis (15). Details of cardiac clinical presentation and management history of 27 patients have been previously published (16). Here, we specifically analyzed the relationship between dietary supplements including B vitamins and “cardiac crises” in patients with TDD. The incidence of cardiac crises in 80 patients (a total of 1,056 person-years) is shown in Figure 4. Among these 80 patients, 32 developed a cardiac crisis at a median age of 4.7 years (IQR, 1.5–7.4 years; range, 4.8 months to 27 years). ECGs during cardiac crises demonstrated prolongation of the corrected QT interval (median value, 547 msec; IQR, 504–600 msec). Cardiac arrest occurred in 22 patients. Of the 12 patients who did not survive the cardiac arrest, 6 cardiac arrests were directly related to uncontrolled arrhythmias. While folate appears to prevent cardiac crises, its effect did not reach statistical significance due to the small number of individuals taking only folate as a supplement. It was also notable that not all B vitamins or supplements appeared to be effective. Patients taking thiamine (vitamin B1), riboflavin (vitamin B2), L-carnitine, or CoQ10 alone continued to have cardiac crisis (Figure 4).

Anecdotally, we identified several patients surviving to adulthood without history of cardiac crisis and not taking supplemental B vitamins. These adult patients came to our attention as the average age of first cardiac crisis is around 5 years and nearly all patients not taking vitamin supplementation had suffered crises by the age of 10 (15, 16). These few adult patients were either taking supplemental folate or were on a diet extremely high in raw leafy green vegetables (a main source of dietary folate). Based on these observational data, we postulate that folate may be a critical B vitamin preventing cardiac crisis and arrhythmias in TDD. Interestingly, folate has been reported to improve both mitochondrial function (22) and ER stress response (23) in murine cardiomyocytes, processes that may be impaired due to loss of TANGO2 function (2, 3, 14). Thus, folate was chosen as the primary effective component in B complex to be tested in our iPSC-CM model.

### Folate ameliorates arrhythmias in patient-derived TANGO2-deficient iPSC-CMs.

**Figure 5:**
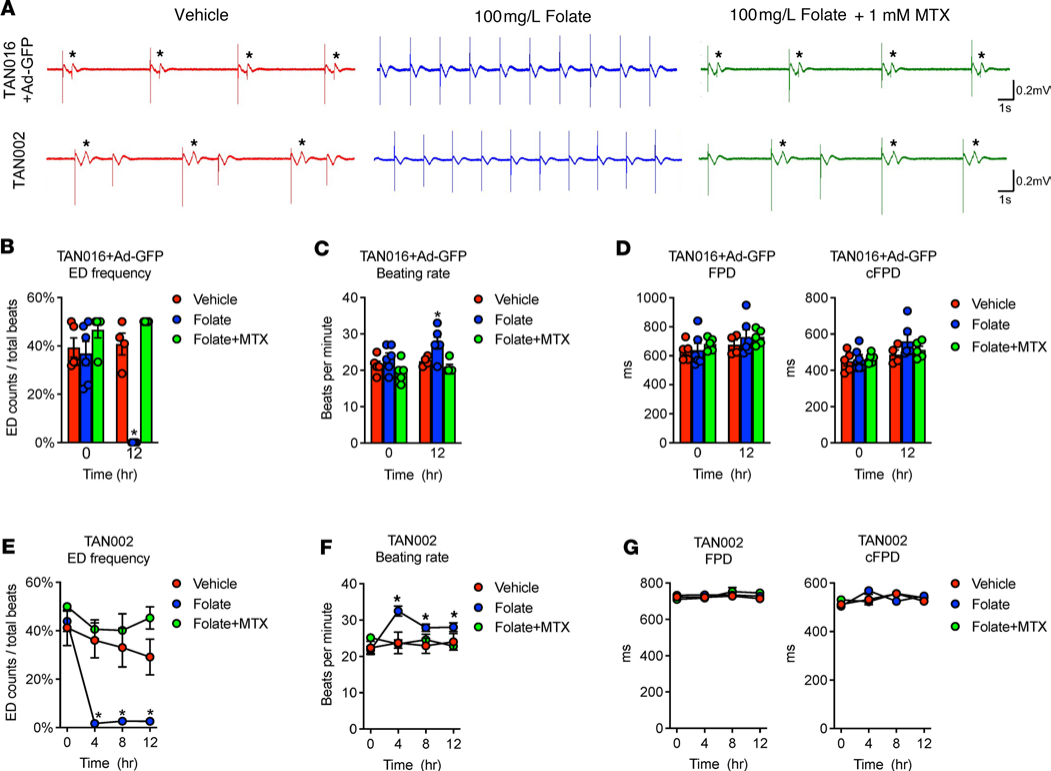
Folate ameliorated arrhythmias in TDD iPSC-CMs. (**A**) Representative field potential recordings of TAN016+Ad-GFP (top row) or TAN002 (bottom row) with vehicle (left), 100 mg/L folate (middle), or folate with 1 mM MTX (right) for 12 hours. In the MTX+folate group, cells were pretreated with 1 mM MTX for 24 hours before applying folate. Asterisk indicates ED. (**B**) ED frequency of TAN016+Ad-GFP with vehicle, folate, or folate with MTX (Folate+MTX) (*n* = 4–6). *P < 0.05 versus vehicle. (**C**) Beating rate of TAN016+Ad-GFP with vehicle, folate, or folate+MTX (*n* = 4–6). *P < 0.05 versus vehicle. (**D**) FPD (left panel) and cFPD (right panel) of TAN016+Ad-GFP with vehicle, folate, or folate+MTX (*n* = 4–6). (**E**) ED frequency in TAN002 with vehicle, folate, or folate+MTX (*n* = 7–43). *P < 0.05 versus vehicle. (**F**) Beating rate of TAN002 and TAN002c vehicle, folate, or folate+MTX (*n* = 7–43). *P < 0.05 versus 0 hours. (**G**) FPD (left panel) and cFPD (right panel) of TAN002 and TAN002c with vehicle, folate, or folate+MTX (*n* = 7–11). Statistical difference was determined by 2-way ANOVA corrected by Šidák method (α = 0.05). Data are mean ± SEM. Error bars smaller than the symbol size are not shown.

We first chose the TAN016+Ad-GFP iPSC-CMs with frequent EDs to monitor the effect of folate treatment in real time. Reduced folate carrier (RFC) is the major transporter for folate uptake into cells (24). At a physiological pH, RFC has a 200- to 400-fold lower affinity for folate in the medium (folic acid; K_m_ = 200–400 μM) than the reduced form of folate (5-methyltetrahydrofolate [5-MTHF]; K_m_ = 1 μM) present in human serum (24–28). Thus, the folate level in the RPMI culture medium (1 mg/L folic acid) is equivalent to the physiological plasma level (>2 ng/mL 5-MTHF) (29). We calculated that a folate concentration of 100 mg/L in vitro approximates the reported serum folate level upon supplementation in patients (160 ng/mL 5-MTHF is approximately 80× plasma level) (30, 31). We found that a single dose of 100 mg/L folate in the culture medium greatly ameliorated the occurrence of EDs, with the effect lasting for at least 12 hours (Figure 5, A and B). Interestingly, we also noted that the amelioration of EDs by folate treatment was associated with a trend of increasing beating rate only in the TANGO2-deficient cells (Figure 5C). Furthermore, pretreatment with 1 mM methotrexate (MTX) — a dose that ensures complete inhibition of dihydrofolate reductase for blocking the folate cycle in the presence of 100 mg/L folic acid (32) — abolished the effect of folate on both EDs and beating rate (Figure 5, A–C). Treatment with folate or folate+MTX did not affect FPD compared with the vehicle group (Figure 5D). Notably, the slight trend of increase in cFPD at 12 hours by folate resulted from an elevation in the beating rate (Figure 5C), not changes in FPD (Figure 5D). In addition, no apparent effect on beating rate, FPD, and cFPD in TAN016+Ad-WT iPSC-CMs was observed when treated with vehicle, folate, or folate+MTX (Supplemental Figure 5, A and B). These findings support that folate exerts its antiarrhythmic effect intracellularly in TANGO2-deficient iPSC-CMs and that the increased beating rate may be associated with its effect.

To validate that the effect of folate is associated with TDD and not specific to TAN016, we next tested the effect of folate on TAN002 iPSC-CMs, which harbor the G154R mutation and have a different genetic background from TAN016 (Figure 5A, bottom panels). Consistent with the observation from TAN016 cells, a single dose of folate reduced ED burden in TAN002 iPSC-CMs to almost zero after 4 hours of folate treatment (Figure 5E). Similar to our findings in TAN016, we observed a significant increase in the beating rate of TAN002 iPSC-CMs from 4 to 12 hours after administration of folate compared with the baseline level at 0 hours (Figure 5F), which was not observed in TAN002c iPSC-CMs that expresses WT-TANGO2 (Supplemental Figure 5C). Importantly, pretreatment with MTX completely abolished both the effects of folate on ED frequency and beating rate (Figure 5, E and F). It is possible that the beneficial effect from folate was associated with the increase in beating rate in TANGO2-deficient iPSC-CMs. Interestingly, these findings support anecdotal clinical observations. While typical antiarrhythmic therapies fail to suppress TDD-related ventricular arrhythmias, rapid atrial pacing and administration of isoproterenol, a β-1 receptor agonist that increases heart rate, have shown transient beneficial effect in treating isolated cases of TDD cardiac crises (16, 33). In addition, FPD and FPDc were not changed by the folate or folate+MTX treatment in either TAN002 or TAN002c iPSC-CMs (Figure 5G and Supplemental Figure 5D). Our results suggest that, while folate drastically reduces arrhythmia events, it may not eliminate the proarrhythmia substrate (QTc prolongation) in patients with TDD. This remains to be further validated by detailed electrophysiology studies.

A recent study showed pantothenic acid Vitamin B5 (B5) rescued the behavioral deficits as well as survival in a TANGO2-deficient *Drosophila* model. Moreover, B5 also rescued the trafficking defect observed in patients with TDD’ dermal fibroblasts (34). We treated both TAN016+Ad-GFP and TAN002 iPSC-CMs with 2 mM B5, the same dose that was effective in human fibroblast in the previous study (34). However, it failed to terminate the ED and did not change the beating rate, FPD, or cFPD in either of the TDD iPSC-CM lines (Supplemental Figure 4). B5 also had no significant effect on any of the isogenic WT iPSC-CM lines, except a mild increase in beating rate of TAN016+Ad-WT (Supplemental Figure 5, E–H). These data likely suggest that multiple B vitamins may be beneficial for patients with TDD and folate is especially important for TDD cardiac crises.

### The antiarrhythmic effect of folate is unrelated to OXPHOS and Ca^2+^ handling capacity in TDD iPSC-CMs at baseline.

Next, we explored the potential mechanism by which folate may mediate the antiarrythmic effect for patients with TDD. To determine whether the TANGO2-deficient iPSC-CMs may have folate deficiency at baseline, we first compared the expression of all 5 plasma folate transporters. Interestingly, we found that the 2 TDD iPSC-CM lines expressed all 5 transporters at a comparable or even higher mRNA level (*FLOR2* in TAN016+Ad-GFP and *FLOR1* in TAN002) compared with that of the isogenic WT lines (Supplemental Figure 6A). We then performed a direct measurement of intracellular folic acid level. Although the baseline levels between the 2 TDD iPSC-CM lines appear to be different, presumably due to different genetic background and potential effects from adenoviral infection on TAN016, no significant difference was found when compared with their isogenic WT controls (Supplemental Figure 6B). Collectively, these data indicate that the TDD iPSC-CMs do not have folate deficiency at baseline, which is consistent with clinical observation.

It has been controversial whether TANGO2 deficiency leads to mitochondrial dysfunction (2, 5, 13, 14, 17). The inconsistent results from different studies likely reflect the large variation among patient-derived dermal fibroblasts (35). Taking advantage of our isogenic iPSC-CM model, we investigated whether TANGO2 deficiency leads to mitochondrial dysfunction, which may be mitigated by folate in cardiomyocytes. The mitochondrial OXPHOS function of isogenic iPSC-CM lines were interrogated by the Seahorse Cell Mito Stress Assay. By comparing the TDD iPSC-CM with the isogenic WT line, we found no significant alterations in either the basal oxygen consumption rate (OCR) or maximal OCR, suggesting a normal OXPHOS function in iPSC-CMs with TANGO2 deficiency (Supplemental Figure 6, C–F). Moreover, folate treatment had no effect on the basal or maximal OCR in either the TDD or isogenic WT iPSC-CM lines. Therefore, the antiarrhythmic effect of folate is not related to mitochondrial OXPHOS function.

Abnormal intracellular Ca^2+^ dynamics has been recognized as an important contributor to cardiac arrhythmias (36). We therefore examined the intracellular Ca^2+^ transient kinetics using Cal-590 AM on an IonOptix recording system with 1 Hz steady-state pacing (37). During the entire period of recording, all the TDD and isogenic WT iPSC-CM lines showed normal Ca^2+^ transient with no rhythmic irregularity, indicating the maturity of our iPSC-CMs (Supplemental Figure 6, G and I). We further analyzed the Ca^2+^ transient duration (50% and 80%) and peak (Supplemental Figure 6, H and J). There is little difference between TAN016 Ad-LacZ (described in Methods) and Ad-WT, and there is no effect of folate on the calcium transient in this isogenic pair. TAN002 showed increased calcium transient peak and slightly increased calcium transient duration than TAN002c, which may be consistent with prolonged cFPD. However, folate does not have any effect in the TAN002 cells on these parameters and, instead, increased calcium transient duration in the TAN002c cells, which is not observed in TAN016 with ad-WT. Folate also had no effect on calcium peak. As a result, we did not observe a consistent calcium transient abnormality in TANGO2-deficient cells that can explain arrhythmic behavior, at least when they are in 1 Hz steady-state pacing condition. Folate treatment also did not produce major alterations on calcium transient in either TANGO2-deficient or control cells. The differences we observed are unlikely to be associated with either the pathophysiology of TANGO2 deficiency or folate’s effect on arrhythmia in TANGO2 deficiency; they are more likely to be attributed to the different genetic backgrounds in TAN016 and TAN002.

We found that TDD iPSC-CMs do not have a folate deficiency, and we have not observed a folate effect on mitochondria OXPHOS or Ca^2+^ handling.

## Discussion

TDD-associated arrhythmias represent one of the most challenging of arrhythmia disorders due to a poor response to standard antiarrhythmic treatment (2, 3) resulting in high mortality (~37%) at a median age of death at 6.5 years (16). To date, no human cardiac model system to study TDD has been reported, to our knowledge, which greatly hinders the investigation and understanding of TDD-associated arrhythmias (5, 34).

In this translational study, we established patient-derived iPSC-CMs from 2 independent genetic backgrounds that carry either a homozygous deletion of exons 3–9 (ΔE3–9; TAN016) or missense variant G154R (TAN002) (Figure 2). Together, these 2 variants account for the majority (around 60%) of all known TDD-affected patients, to date (15). The 2 TANGO2-deficient iPSC-CMs lines successfully recapitulated key arrhythmic phenotypes in TDD, including prolonged QT intervals and frequent EDs, both of which were abolished in isogenic control lines either by expression of WT-TANGO2 using an adenoviral vector or CRISPR-mediated correction of the missense variant.

Our natural history study of 80 patients with TDD suggests 1 or multiple B vitamins may be associated with reduced risks for cardiac crises in patients with TDD (Figure 4). Using our novel cardiac models, we demonstrated that high-dose folate virtually abolished the EDs in both TAN016 and TAN002 iPSC-CM cell lines and eliminated pauses in TAN002 (Figure 5). We observed that the elimination of EDs by folate coincides with an increase in beating rate in the TANGO2-deficient iPSC-CMs. Importantly, MTX blocked the antiarrhythmic effect of folate, suggesting that intracellular folate mediates this effect. These results provide the first direct experimental evidence to our knowledge supporting the beneficial effect of folate for TANGO2 deficiency associated arrhythmia. Folate has never been reported to have an antiarrhythmic or proarrhythmic effect. In our study, its antiarrhythmic effect seems to be specific to TANGO2-deficient cells; however, it may have broader implications in other arrhythmias associated with genes commonly implicated in energy or metabolic processes, such as *TECRL* (38), *EXOSC5* (39), and *C1QBP* (40). Our study may also have implication on the 22q11.2 deletion syndrome where the region of recurrent microdeletions may include the part of the whole TANGO2 gene body in certain patients (7, 41, 42). The exact molecular function of folate in TANGO2 deficiency remains unknown, and we are actively pursuing this direction. Because it only takes folate 4 hours or even less time to terminate the arrhythmias (Figure 5E), we speculate that the underlying mechanism might be a direct regulation of cardiac ion channels. Folate participates in one-carbon metabolism, which provides methyl group for protein posttranslational modification (43). Thus, it may be possible that the cardiac ion channel methylation profile is altered by folate within a short period of time, and this is known to cause dramatic change in ion channel function (44, 45). A more comprehensive characterization of the posttranslational modification profile of key cardiac ion channels using mass spectrometry in future studies may test this hypothesis.

### Total parental nutrition containing folate may have terminated arrhythmic storm in 1 patient with TDD.

**Figure 6:**
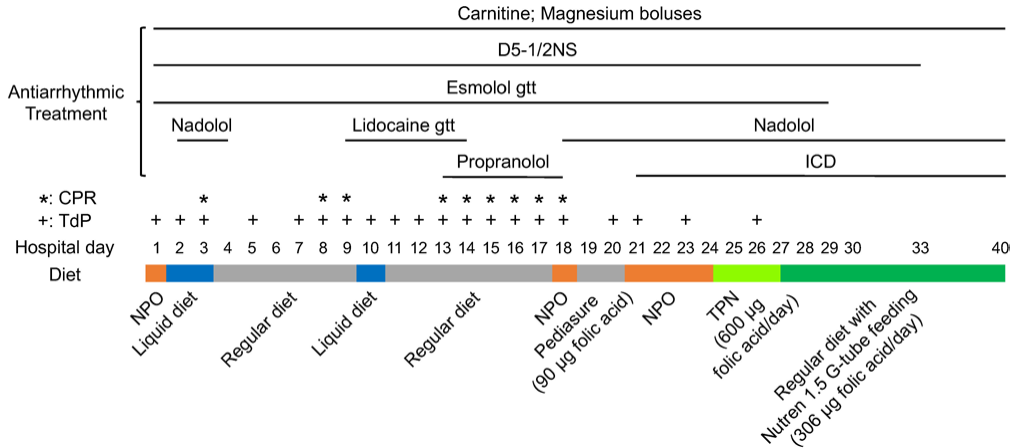
Timeline of the 13-year-old patient admitted during a cardiac crisis. Arrhythmias were resolved within 37 hours of starting folate containing TPN.

To substantiate our finding in iPSC-CMs derived from patients with TDD with clinical evidence, we conducted a detailed chart review of a 13-year-old Hispanic female carrying homozygous G154R variant, who had a prolonged hospital stay due to TDD cardiac crises during which she suffered multiple cardiac arrests secondary to TdP (Figure 6). Over the course of 26 days, she developed TdP on 20 days and required CPR on 9 of these days. She was treated with dextrose, carnitine, esmolol, magnesium, nadolol, lidocaine, and propranolol without an effect. During these 26 days, nutritional support was limited. Although a regular diet was ordered, oral intake consisting of liquids and popsicles was poor and interrupted by numerous code events leading to a 10 lb weight loss. On the evening of hospital day 24, she was initiated on total parental nutrition (TPN) containing all B vitamins including 400 μg of daily folate. Within 37 hours of starting folate containing TPN (equivalent to total of 600 μg of folate intake), all arrhythmias resolved. The patient was transitioned to supplemental formula (still containing multivitamins) and discharged home with no further arrhythmias for the next 9 years, to date, while she continued on supplemental formula. While TPN contains full nutrition support and we cannot exclude the role of many other components, this dramatic case supports the use of early nutritional support with vitamin supplementation in patients with TDD with cardiac crises.

### Limitations.

One limitation of the study is that we are not able to perform functional studies during arrhythmia to mimic crises. The arrhythmias in both patients with TDD and their iPSC-CMs were spontaneous and episodic. The arrhythmias were captured by RTCA during about 1% of the total recording time spanning several weeks. Although we could monitor arrhythmias using RTCA, we could not ensure that the cells remained in arrhythmia while performing other functional studies such as Seahorse assay or calcium imaging. Future investigation on the triggers for TDD-associated arrhythmias will be essential to resolve this issue.

### Conclusion.

We believe our patient-derived iPSC-CMs model serves as a valuable platform for further investigating the molecular mechanism of cardiac arrhythmias and for identifying novel therapies for TDD. B vitamins may help reduce the risk for metabolic crises, and in particular, folates may be useful in terminating lethal cardiac arrhythmias in patients with TDD. Folate is a water-soluble vitamin supplement with negligible side effects at physiological levels and, thus, can serve as a potentially life-saving treatment or preventive strategy for the highly lethal TDD-associated arrhythmias. The detailed antiarrhythmic mechanism of folate in TANGO2-deficient cardiomyocytes requires future investigation.

## Methods

### Sex as a biological variable.

Our study examined male and female patients, and similar findings were reported in both.

### Natural history study.

Data from 80 patients with TDD was collected using questionnaire-based interviews conducted by video or phone with interpreters as necessary and using available medical records including metabolic crisis and supplemental vitamin intake. Metabolic crisis was defined as a hospital admission associated with rhabdomyolysis and elevated creatine kinase (CK) above normal range. Cardiac crisis was defined as the development of ventricular arrhythmias, cardiomyopathy, or cardiac arrest during a metabolic crisis. Incidence rates (IR) were computed by dividing the number of cardiac crises in each group by the sum of person-years in which subjects were taking the vitamin. Rate differences (RD) were computed by subtracting IR among those taking the vitamin from the IR among those not taking it (IR_off_ _vitamin_ – IR_on_ _vitamin_).

### Patient information.

Both patients are part of the natural history study.TAN016 is currently an 8-year-old non-Hispanic White female with a history of 3 metabolic crisis between the ages of 4 and 6 months of age. During her crisis, she developed prolonged QTc and cardiac systolic dysfunction. Since starting supplemental formula that contains all B vitamins including folate, she has not had a subsequent crisis.

TAN002 is currently an 11-year-old Hispanic male with a history of 3 metabolic crises at the age of 7 years. During his crises, he developed prolonged QTc, type I Brugada pattern, systolic dysfunction, and life-threatening ventricular arrhythmias that responded to isoproterenol. He has not had any recurrent crisis since being initiated on supplemental formula and B complex vitamins containing folate.

### Generation and maintenance of iPSCs.

iPSC reprogramming was performed by Human Stem Cell Core in Baylor College of Medicine following a published protocol (46). Slight modification was made where ReproTeSR Medium (Stemcell Technologies, 05926) was used during the first week of culture and then switched to mTeSR 1 (Stemcell Technologies, 85850) for initial expansion. Primary skin fibroblasts from a 7-month-old and 8-year-old patients with TDD (at the time of collection) were used to generate TAN016 and TAN002 iPSC lines, respectively. TAN002c was generated from TAN002 by CRPISR editing. The control iPSC line was reprogrammed using peripheral blood mononuclear cells (PBMCs) (47) from a 37-year-old healthy female donor, and it has been registered as BCMi002-A in hPSCreg (https://hpscreg.eu/). All iPSC lines have passed quality control for pluripotency test, karyotyping, and mycoplasma test.

iPSCs were seeded on surface coated with hESC-Qualified Matrigel (Corning, 354277) and maintained in mTeSR 1 or mTeSR Plus medium (Stemcell Technologies, 100-0276). iPSCs were dissociated with ReLeSR (Stemcell Technologies, 05872) or Gentle Cell Dissociation Reagent (Stemcell Technologies, 100-0485) for regular passage in cell clumps. For passage in single cells, iPSCs were dissociated with Accutase (Innovative Cell Technologies, AT104) or Accumax (Innovative Cell Technologies, AM105) and were seeded in medium supplemented with 10 μM Y-27632 (MedChemExpress, HY-10071). Y-27632 was removed from the medium after 24 hours.

### Generation of TAN002c by CRISPR editing.

In total, 500,000 singularized TAN002 iPSCs were resuspended in the Lonza nucleofection P3 primary cell solution (Lonza, V4XP-3024), with 100 picomoles of sgRNA (Synthego), 20 picomoles of Alt-R S.p. HiFi Cas9 Nuclease V3 (Integrated DNA Technologies, 1081060), and 2 μg of template ssODN (Integrated DNA Technologies) for homology directed repair. RNP was allowed to form in P3 solution for at least 5 minutes at room temperature, and the nucleofection mixture was loaded into the 16-well nucleocuvette with the total volume less than 25 μL. Electroporation was performed in the 4D-Nucleofector X Unit (Lonza, AAF-1003X) using program CB-150 or CA-137. iPSCs were immediately transferred into culture medium supplemented with 10 μM Y-27632 after nucleofection. Individual colonies were subsequently isolated for genotyping and quality check. Sequences of sgRNA and ssODN used are listed below: sgRNA: 5′-CAGCGCGUUGCUCAGCCUGU-3′; template ssODN for homology directed repair: 5′-CTCTGCATGGCCCGCTGATTGCTCCTCACAGGCACCTACGGGCTGAGCAACGCGCTGCTGGAGACTCCCTGGAGGAAGCT-3′.

### Differentiation, maintenance, and expansion of iPSC-CMs.

Two differentiation protocols were used for this study. iPSC-CMs used for experiments related to Figure 2, B–F, were generated by STEMdiff Ventricular Cardiomyocyte Differentiation Kit (Stemcell Technologies, 05010), and the rest were all generated by the GiWi method. When using GiWi method, we follow published protocols (48, 49). Briefly, for induction on day 0, medium was changed to RPMI1640 (Thermo Fisher Scientific, 11875119) with B27 supplement minus insulin (Thermo Fisher Scientific, 17504001) and 8 or 10 μM CHIR99021 (Cayman Chemical, 13122). On day 1, medium was changed to RPMI1640+B27 minus insulin. On day 3, half volume of the medium was changed to RPMI1640+B27 minus insulin supplemented with a final concentration of 5 μM IWP-2 (Cayman Chemical, 13951). On day 5, medium was changed to RPMI1640+B27 minus insulin. Starting from day 7, RPMI1640 with B27 supplement (Thermo Fisher Scientific, 17504001) was used for maintenance of iPSC-CMs and changed every 3 days. Spontaneous contraction was typically observed on day 7. iPSC-CMs were further enriched by lactate selection using glucose-free RPMI1640 (Thermo Fisher Scientific, 11879020) supplemented with 4 mM lactate (MilliporeSigma, L7022) from day 20 to 24 (19). When iPSC-CMs were generated using STEMdiff Ventricular Cardiomyocyte Differentiation Kit, we follow the instructions from the manufacture and achieved similar differentiation efficiency (over 85%). Experiments with iPSC-CMs were conducted between day 50 and day 90 after differentiation. For genotyping, iPSC-CMs were further expanded by RPMI1670+B27 supplemented with 2 μM CHIR99021 (50). Expansion was terminated by removal of CHIR99021 in the medium.

### Generation of adenovirus.

Adenoviruses expressing WT-TANGO2, G154R-TANGO2, and EGFP were generated and tittered by VectorBuilder. Ad-GFP was used as infection control for most of the experiments. For intracellular folic acid level measurement and Ca^2+^ imaging, adenovirus expressing β-galactosidase (Ad-lacZ) (Welgen Inc.) was used as infection control to avoid possible interference from fluorescent protein. The viral construct contains coding sequence of WT or G154R-hTANGO2 with 3XFLAG tag at the C-terminus. The expression was driven under an EF-1α promoter. Adenovirus infection on iPSC-CMs was performed with the minimal dose to achieve 100% infection for all experiments. The 100% infection rate was demonstrated by 100% GFP positive cells at 4 days after Ad-GFP infection in TAN016 iPSC-CMs with nuclear counterstain by 2 μg/mL Hoechst 33342 (Thermo Fisher Scientific, H3570).

### DNA extraction, RNA extraction, and reverse transcription.

Genomic DNA was extracted using QIAamp DNA Mini Kit (Qiagen, 51304). RNA was extracted using the High Pure RNA Isolation Kit (Roche, 11828665001). Reverse transcription was performed using iScript Reverse Transcription Supermix (Bio-Rad, 1708841).

### qPCR.

Real-time TaqMan-qPCR was assembled in qPCRBIO Probe Blue Mix (Genesee Scientific Corporation, 17-514) with 0.5 μM of each primer (Integrated DNA Technologies) and 50 nM Universal ProbeLibrary Probe (Roche) for quantifying the gene expression of *MYL2*, *MYH7*, *MYH6*, *SCN5A*, *KCNIP2*, *RYR2*, *ATP2A2*, and *PPIB*. For quantifying the expression of *KCNJ2* (51), *SLC19A1* (52), *SLC46A1* (53), *FOLR1* (54), *FOLR2* (54), and *FOLR3* (55), iTaq Universal SYBR Green Supermix was used (Bio-rad Laboratories, 1725121) for the RT-PCR master mix. qPCR was performed in QuantStudio 5 Real-Time PCR System (Thermo Fisher Scientific) and calculated using 2^–ΔΔC^ method. The expression level of *PPIB* was used as internal control unless otherwise indicated. qPCR primer sequences are provided in Supplemental Table 1.

### Genotyping and Sanger sequencing.

Primer pairs targeting exon 1–2 (forward: 5′-CAGGCTGCTTGAAGACCTCG-3′; reverse: 5′-ACGCGTTTTTGGAAACAGGG-3′) and exon 7–8 (forward: 5′-CAACAATGAAGAGGCGCAGC-3′; reverse: 5′-GTACTTGCTCAGCATGGGCTG-3′) of human *TANGO2* transcript (ENSG00000183597) were used to genotype the deletion mutation from exon 3–9 deletion in TAN016 using RT-PCR. cDNA from TAN016 and the WT iPSC-CMs (BCMi002-A) were used as templates for PCR. The PCR products were separated in a 2% agarose gel and imaged by ChemiDoc Touch Imaging System (Bio-rad Laboratories). Detections of exon 1–2 and exon 7–8 were indicated by 105 bp and 82 bp amplicons, respectively.

Sanger sequencing was performed to confirm the G154R mutation in TAN002 and correction in TAN002c. PCR was carried out in Platinum SuperFi II Green PCR Master Mix (Thermo Fisher Scientific, 12369010) with a primer pair (forward: 5′-TCTCCTTGCCATGCCATCAG-3′; reverse: 5′-CCCACTCACGCCTCTTCATT-3′) to amplify a region containing the G154R/correction site from the genomic DNA. The PCR product was purified by PCR purification kit (Zymo Research, D4013) and sent to Azenta Life Sciences for Sanger sequencing. PCR primers along with 2 additional internal primers (5′-CTGGCAGCACTCACCAACTA-3′; 5′-CAGCTGCGCCTCTTCATTG-3′) were used as sequencing primer to ensure specificity. All primers used in the study were synthesized by Integrated DNA Technologies.

### Immunoblotting.

We lysed iPSC-CMs in RIPA buffer (Thermo Fisher Scientific, 89901) supplemented with protease inhibitor cocktail (Roche, 11836153001). Protein concentration was determined using BCA assay kit (Thermo Fisher Scientific, 23225). Protein samples were mixed with LDS sample buffer (Thermo Fisher Scientific, B0008) and reducing agent (Thermo Fisher Scientific, B0009) and were then incubated at 70°C for 10 minutes. Proteins were separated on Bolt 4%–12% Bis-Tris gels (Thermo Fisher Scientific, NW04122BOX) with MES SDS running buffer (Thermo Fisher Scientific, B0002). Resolved proteins were then transferred to 0.2 μm PVDF membrane (Bio-Rad, 1704272) by semi-dry transfer at 25V (up to 1A) for 30 minutes using Trans-Blot Turbo Transfer System (Bio-Rad, 1704150). PVDF membrane was then blocked with 5% nonfat milk (Bio-Rad, 1706404) in tris-buffered saline with 0.1% Tween (TBST) for 1 hour at room temperature. The membrane was then incubated with primary antibody at 4°C overnight and then with mouse anti–rabbit IgG-HRP secondary antibody (Santa Cruz Biotechnology, sc-2357) at room temperature for 2 hours. Enhanced chemiluminescence substrate of HRP (Thermo Fisher Scientific, 34580 or 34094) was used for detection. Na^+^/K^+^ ATPase (NKA) was used as internal control to avoid possible overlapping with the TANGO2 bands. Primary antibodies used are listed below: anti-TANGO2 (Proteintech, 27846-1-AP) (13), anti-NKA (Cell Signaling Technologies, 3010), and anti-FLAG (Cell Signaling Technologies, 14793). All were used at 1:1,000 dilution.

### Immunofluorescence staining.

Human iPSC-CMs were seeded at 25,000 per well in 8-well Nunc Lab-Tek II Chamber Slide (Thermo Fisher Scientific, 154453) coated with 2.5 μg/cm^2^ of fibronectin (MilliporeSigma, FC010). Medium was replaced with RPMI1640+B27 two days after seeding for maintenance until further usage. Cells were fixed by 4% PFA in PBS at room temperature for 15 minutes and permeabilized with 0.2% Triton-X in PBS at room temperature for 20 minutes. Slides were then blocked with 5% horse serum (Thermo Fisher Scientific, 16050130) in PBS at room temperature for 2 hours. Slides were further incubated with primary antibody in 2.5% horse serum PBS at 4°C overnight and were then incubated with goat anti–mouse IgG H&L–conjugated with Alexa Fluor 594 (Abcam, ab150116) along with DAPI (Thermo Fisher Scientific, D3571) at room temperature for 1 hour. Images were taken using EVOS FL Auto Imaging System (Thermo Fisher Scientific) under a ×20 objective (Thermo Fisher Scientific, AMEP4734) with light cubes for DAPI (Thermo Fisher Scientific, AMEP4650) and Texas Red (Thermo Fisher Scientific, AMEP4655) channels. Primary antibodies used included: anti-α-actinin (MilliporeSigma, A7811) and anti-cTnT (Abcam, ab8295). Primary and secondary antibodies were all used at 1:500 dilution.

### Flow cytometry analysis for cTnT^+^ cells.

iPSC-CMs were harvested with STEMdiff Cardiomyocyte Dissociation Kit (Stemcell Technologies, 05025), stained with LIVE/DEAD Fixable Near IR 780 Viability Kit (Invitrogen, L34992), and then fixed with Fixation/Permeabilization solution (BD Biosciences) for 20 minutes. Cells were subsequently stained with primary antibody (anti-cTnT antibody [1C11], ab8295, 1:200) and secondary antibody (goat anti–mouse IgG H&L [Alexa Fluor 488], 1:500) in 1 × Perm/Wash buffer (BD Biosciences). The cells were analyzed using AttunNXT analyzer. Data were analyzed using FlowJo software (FlowJo).

### Recording and analysis of FP for iPSC-CMs.

We seeded iPSC-CMs at 15,000 per well in 100 μL volume on E-Plate CardioECR 48-well plate (Agilent Technologies, 300601110) coated with fibronectin. After seeding, iPSC-CMs were maintained for at least 2 weeks before any treatment or adenoviral infection. Folic acid (MilliporeSigma, F8758) was freshly prepared at 100 g/L in 1N NaOH (1,000× stock). MTX (MilliporeSigma, PHR1396-1G) was freshly prepared at 500 mM in 1N NaOH (500× stock). D-pantothenic acid sodium salt (Cayman Chemical, 17288) was freshly prepared at 2 mol/L in H_2_O (1,000× stock). All the stock solutions were further diluted to 50× in RPMI1640+B27 for better pipetting accuracy. No change in medium pH was observed after administration of the folic acid or MTX solution. In total, 1 mM NaCl was used as vehicle control. Because the treatment was performed in a laminar hood and outside the incubator, we observed a 3-hour equilibrium time for iPSC-CMs to fully restore the beating rhythm when placed back in the incubator. Therefore, data points between 0 and 3 hours after treatment were not included for analysis.

FP was recorded by xCELLigence RTCA CardioECR (Agilent Technologies) in a 37°C incubator with 5% CO_2_ (20). The duration of each recording was at least 20 seconds. FP electrical signals were recorded by 2 extracellular FP electrodes in each well, in which the one with better signal-to-noise ratio was used for analysis. Beating rate, FPD, and corrected FPD (cFPD) was calculated by the RTCA CardioECR analysis software (Agilent). Fridericia’s formula (FPDc = FPD/interspike interval^1/3^) was adopted for correction to minimize influence of a wide range of interspike interval on FPDs, as was suggested in previous publications (56, 57). FP waveform containing ED event was excluded from FPD and cFPD calculation, since the time from the depolarization to repolarization was unable to define. ED events were counted manually, and the ED frequency was calculated as the percentage of ED counts in total beats in a recording.

### Intracellular folic acid level measurement.

The folic acid level was measured using the folic acid ELISA kit (Abcam, ab285228) and followed the sample preparation protocol for tissue with minor modifications. Briefly, 1 million iPSC-CMs were dissociated and washed with PBS 3 times to eliminate potential folic acid contamination from culture medium. Cells were then resuspended in 100 μL of sample dilutent in which 50 μL was used for ELISA, and 2 μL was used to determine protein concentration using BCA assay kit (Thermo Fisher Scientific, 23225) for normalization. The optical density (OD) was measured in FLUOstar Omega microplate reader and analyzed with the built-in software (BMG LABTECH). Standard curve for folic aicd ELISA measurement was fitted by 4-parameter logistic regression (*R*^2^ > 0.99). The folic acid level was normalized to protein content in each sample.

### Seahorse cell mito stress test.

iPSC-CMs were seeded at 50,000 per well in Matrigel-coated (Corning, 354277) Seahorse XF96 cell culture microplates in RPMI1640 supplemented with 1% B27, 10% KnockOut Serum Replacement (Thermo Fisher Scientific, 10828028), and 10 μM Y-27632 (MedChemExpress, HY-10071). Medium was replaced with RPMI1640+1% B27 2 days after seeding for maintenance until further usage. OCR was measured in extracellular flux analyzer XFe96 (Agilent) with pyruvate (Sigma-Aldrich, P2256) as substrate following the manufacturer’s protocol. In total, 1.5 μM oligomycin (Sigma-Aldrich, 495455), 0.5 μM FCCP (Sigma-Aldrich, C2920), 5 μM rotenone (Sigma-Aldrich, 45656), and 5 μM antimycin A (Sigma-Aldrich, A8674) were used for the assay protocol (58). Quantification of basal and maximal OCR was performed according to the Seahorse XF Cell Mito Stress Test Kit User Guide.

### Ca^2+^ transient measurements.

IPSC-CMs were detached with TrypLE select (Thermo Fisher Scientific, A1217701) and replated into 35 mm dishes with glass-like polymer coverslips (200,000 cells per coverslip). Cells were allowed to recover for 1 week and then treated with 1 mM NaCl as the vehicle control or 100 mg/L folate resuspended in culture media for 4 hours at 37°C. Cal-590-AM (10 μM, AAT Bioquest, 20510) was loaded for 30 minutes at 37°C in Tyrode’s buffer (135 mM NaCl, 5 mM KC1, 1.8 mM CaCl_2_, 1 mM MgSO_4_, 1 mM HEPES, 5.6 mM glucose, pH 7.4). Cells were washed with Tyrode buffer and incubated for 30 additional minutes at room temperature to allow dye deesterification at 37°C. Calcium transients were measured using a dual-wavelength IonOptix equipped with 470 and 565 LEDs. Experiments were conducted at 37°C, during steady-state pacing at 1 Hz, and perfused continuously with Tyrode buffer. Data were analyzed using Cytosolver and Ionwizard (IonOptix).

### Statistics.

All the statistical analyses were performed in GraphPad Prism 9 and were specified in each figure legend. The unpaired 2-tailed Student *t* test was performed for comparison between 2 groups. Either 1-way ANOVA with Dunnett’s correction (α = 0.05) or 2-way ANOVA with Šidák correction (α = 0.05) was performed for comparisons between multiple groups. A *P* value less than 0.05 was considered significant.

### Study approval.

The TDD natural history has been ongoing since February 2019 and is approved by the IRB of Baylor College of Medicine (#H-43240)

### Data availability.

All the raw data values for each graphs are provided in the Supporting Data Values file.

## Author contributions

LZ, CYM, and SRL conceived the project. WX and LZ designed all the experiments. WX, Y Cao, and WP performed all the experiments. WX, WP, ACY, and FA performed data analysis for all the experiments. SBS, MJA, Y Chen, and CYM designed and performed data analysis for the natural history study. JJK generated the iPSC lines. WX, Y Cao, WP, LS, SRL, NL, FTH, FA, XHTW, CYM, and LZ prepared the figures and wrote the manuscript.

## Supplementary Material

Supplemental data

Unedited blot and gel images

Supplemental video 1

Supplemental video 2

Supplemental video 3

Supplemental video 4

Supplemental video 5

Supplemental video 6

Supplemental video 7

Supporting data values

## Version 1 06/10/2024

Electronic publication
