# Supplementary material for: Folate as a potential treatment for lethal ventricular arrhythmias in TANGO2-deficiency disorder
Source: JCI Insight. 2024 Jun 10;9(11):e171005. doi: 10.1172/jci.insight.171005 (PMC11382877; doi:10.1172/jci.insight.171005)
Supplement: Supplemental data [file jciinsight-9-171005-s051.pdf]

Supplemental Figure 1

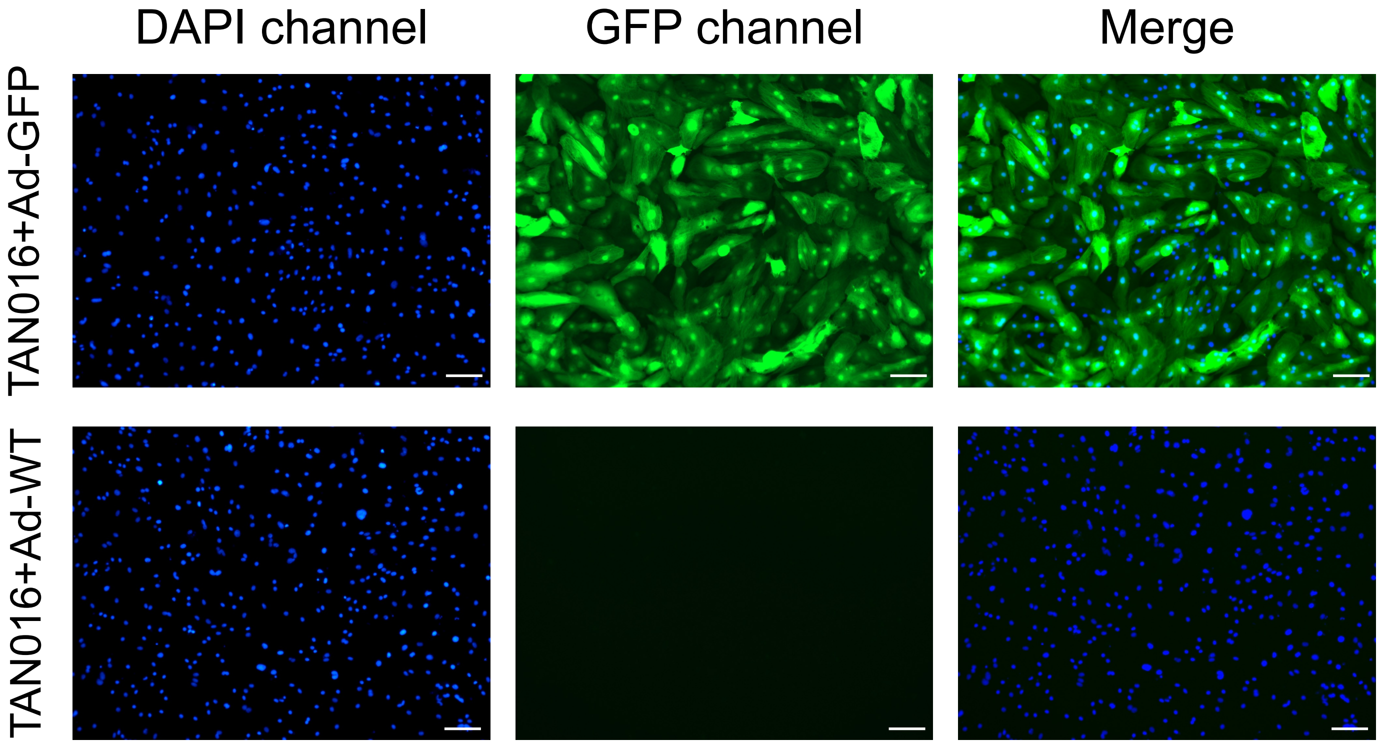

Supplemental Figure 1. Adenoviral Infection efficiency on TAN016 iPSC-CMs. Fluorescent images were taken from TAN016+Ad-GFP or TAN016+WT (negative control) 4 days post infection under the DAPI and GFP channels with the same settings. Hoechst 33342 was used to stain the nuclei in live iPSC-CMs. Scale bars indicate 100 μm.

Supplemental Figure 2

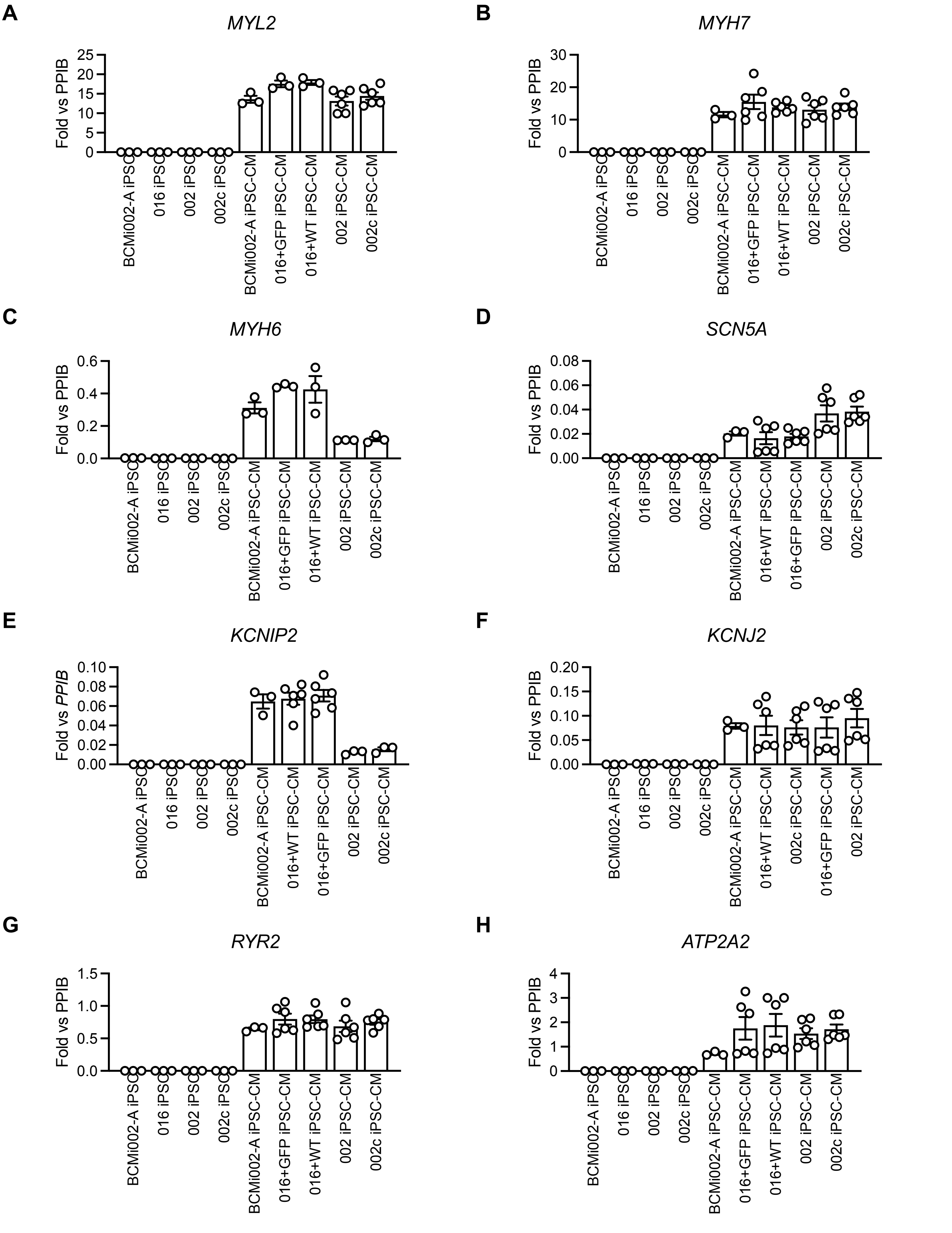

Supplemental Figure 2. Transcriptional level of key cardiac marker genes in 4 iPSC and 5 iPSC-CM lines used in the study. Quantification was performed by RT-PCR and presented as fold change vs *PPIB* (n=3-6). Data are mean + SEM. BCMi002-A iPSC/iPSC-CM was used as an additional independent WT control.

Supplemental Figure 3

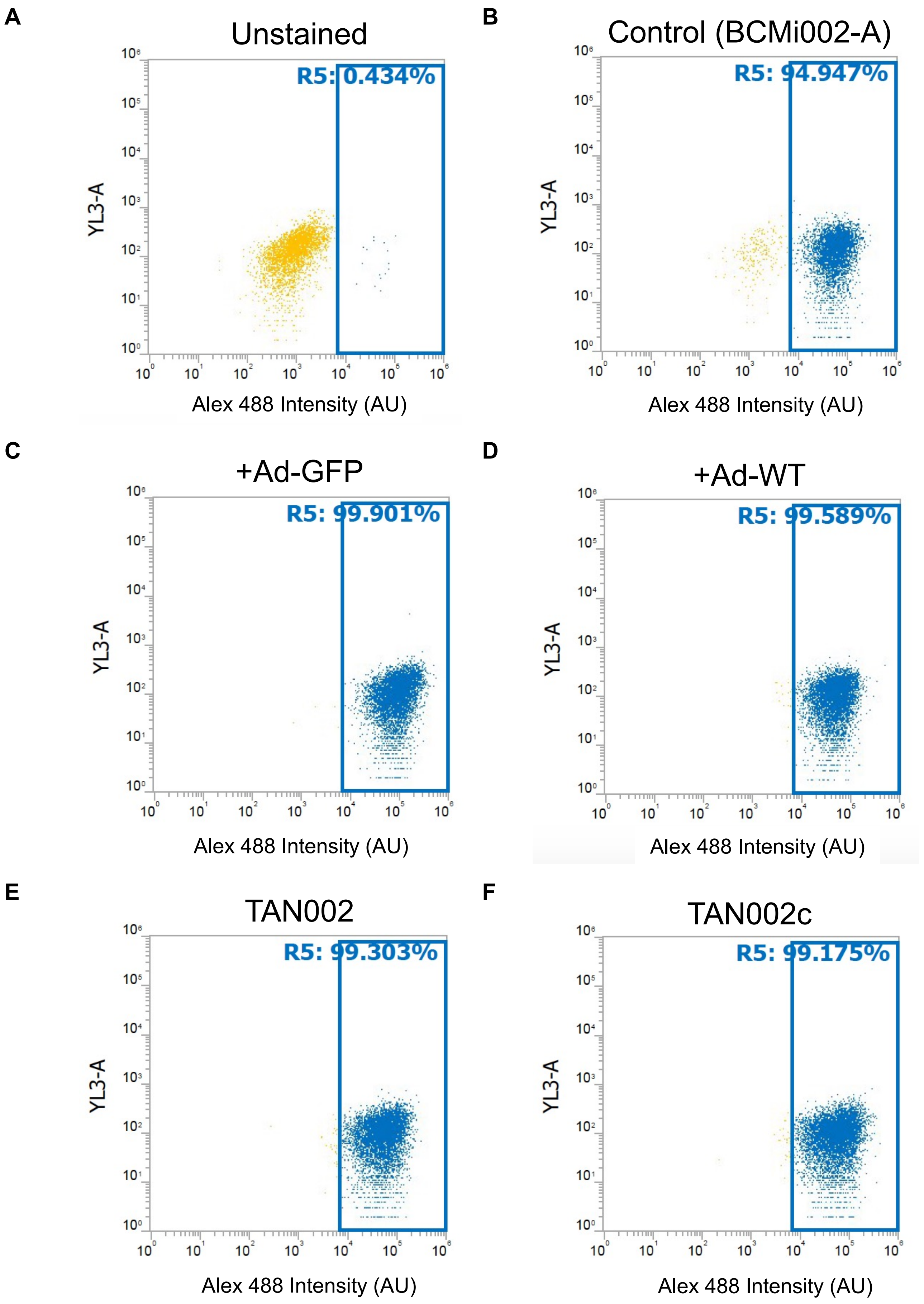

Supplemental Figure 3. Analysis of cTnT-positive cells by flow cytometry. Cells in experimental groups (B-F) were stained with primary antibody against cardiac troponin T (cTnT) and secondary antibody conjugated with Alexa Fluor® 488. Sample without primary antibody staining was used as unstained control for gating (A). Live/dead staining was also performed to exclude dead cells. cTnT-positive population was determined by the fluorescent intensity (arbitrary unit) of the Alexa fluor 488 channel. The percentage of cTnT-positive cells in each sample was shown on the graph.

Supplemental Figure 4

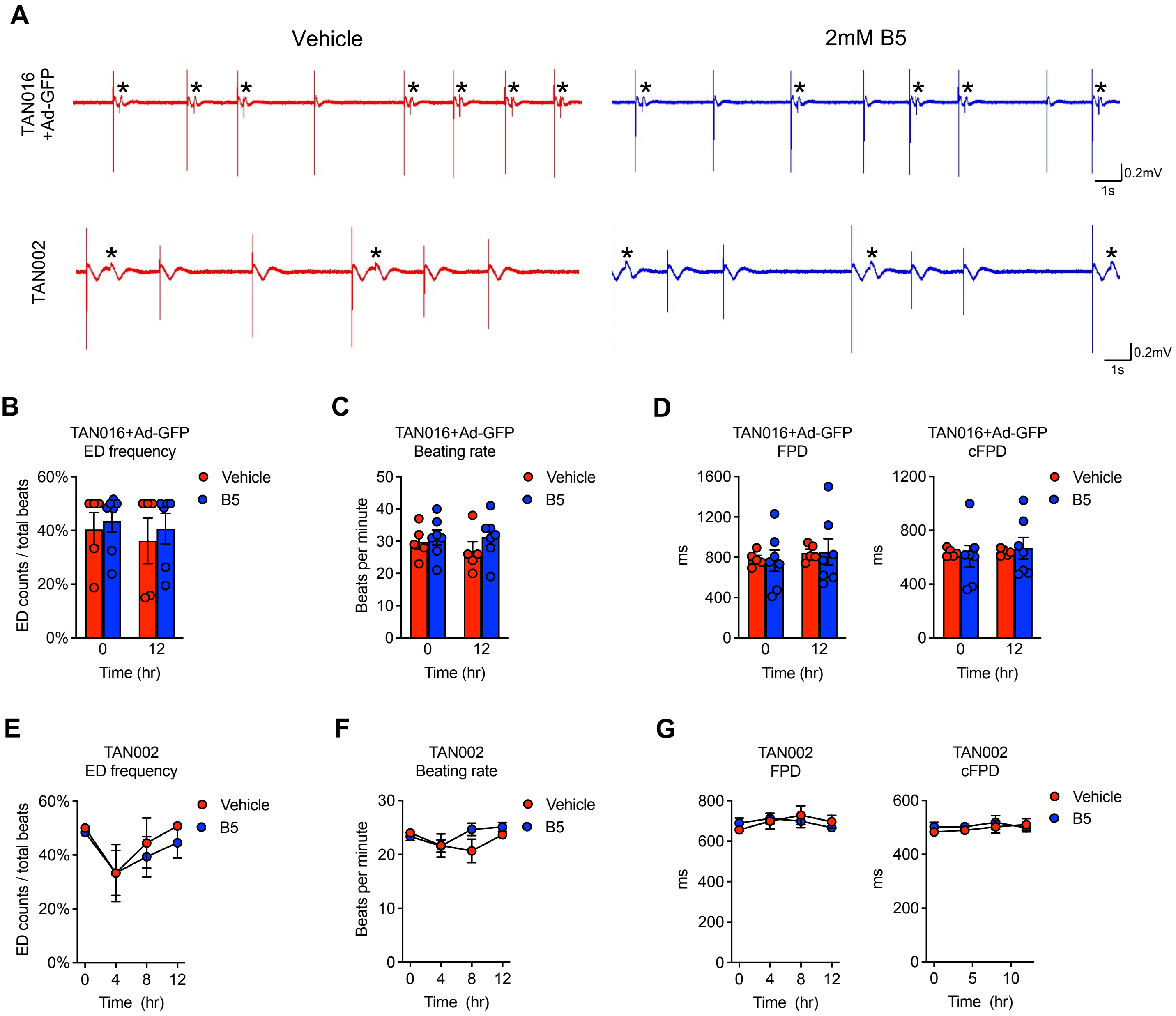

Supplemental Figure 4. Vitamin B5 failed to ameliorate arrhythmias in TDD iPSC-CMs. (**A**) Representative field potential recordings of TAN016+Ad-GFP (top row) or TAN002 (bottom row) with vehicle (left) and 2 mM Vitamin B5 (right) for 12 hrs. *: ED. (**B-D**) ED frequency, beating rate, FPD and FPDc of TAN016+Ad-GFP with vehicle and 2 mM Vitamin B5 (n=5-7). (**E-G**) ED frequency, beating rate, FPD and FPDc of TAN016+Ad-GFP with vehicle and 2 mM B5 (n=6-9). Statistical difference was determined by two-way ANOVA corrected by Sidak method (α=0.05). Data are mean + SEM. Error bars smaller than the symbol size are not shown.

Supplemental Figure 5

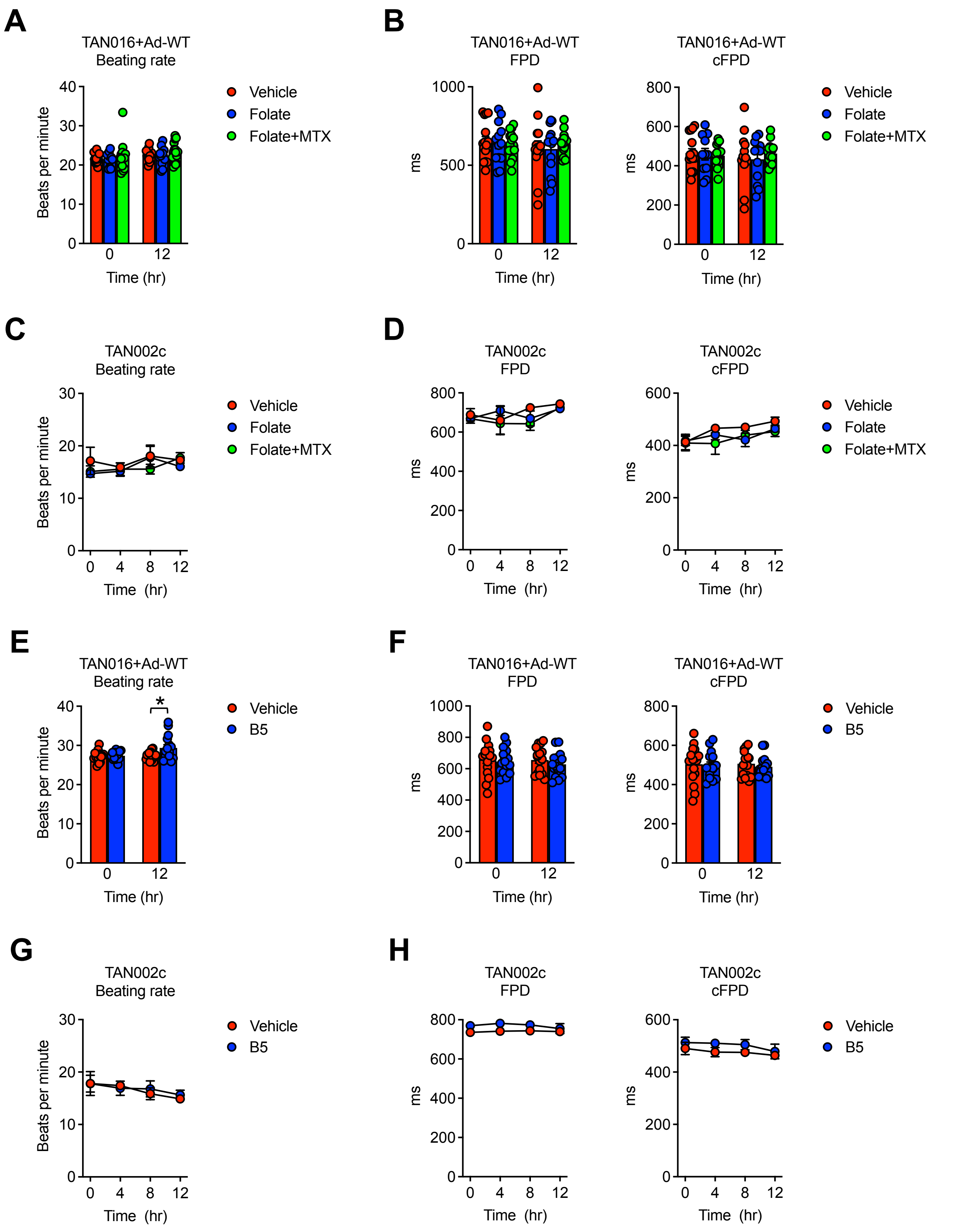

Supplemental Figure 5. Effect of vehicle, folate, folate with MTX, and Vitamin B5 on the beating rate, FPD, and cFPD of isogenic WT iPSC-CMs. (**A and B**) Effect of folate and folate+MTX treatment on the beating rate (A), FPD (left panel in B), and cFPD (right panel in B) of TAN016+Ad-WT iPSC-CMs. (**C and D**) Effect of folate and folate+MTX treatment on the beating rate (C), FPD (left panel in D), and cFPD (right panel in D) of TAN002c iPSC-CMs. (**E and F**) Effect of Vitamin B5 treatment on the beating rate (E), FPD (left panel in F), and cFPD (right panel in F) of TAN016+Ad-WT iPSC-CMs. (**G and H**) Effect of B5 treatment on the beating rate (G), FPD (left panel in H), and cFPD (right panel in H) of TAN002c iPSC-CMs. *: $p<0.05$, two-way ANOVA corrected by Sidak method ($\alpha=0.05$). Data are mean + SEM. Error bars smaller than the symbol size are not shown.

Supplemental Figure 6

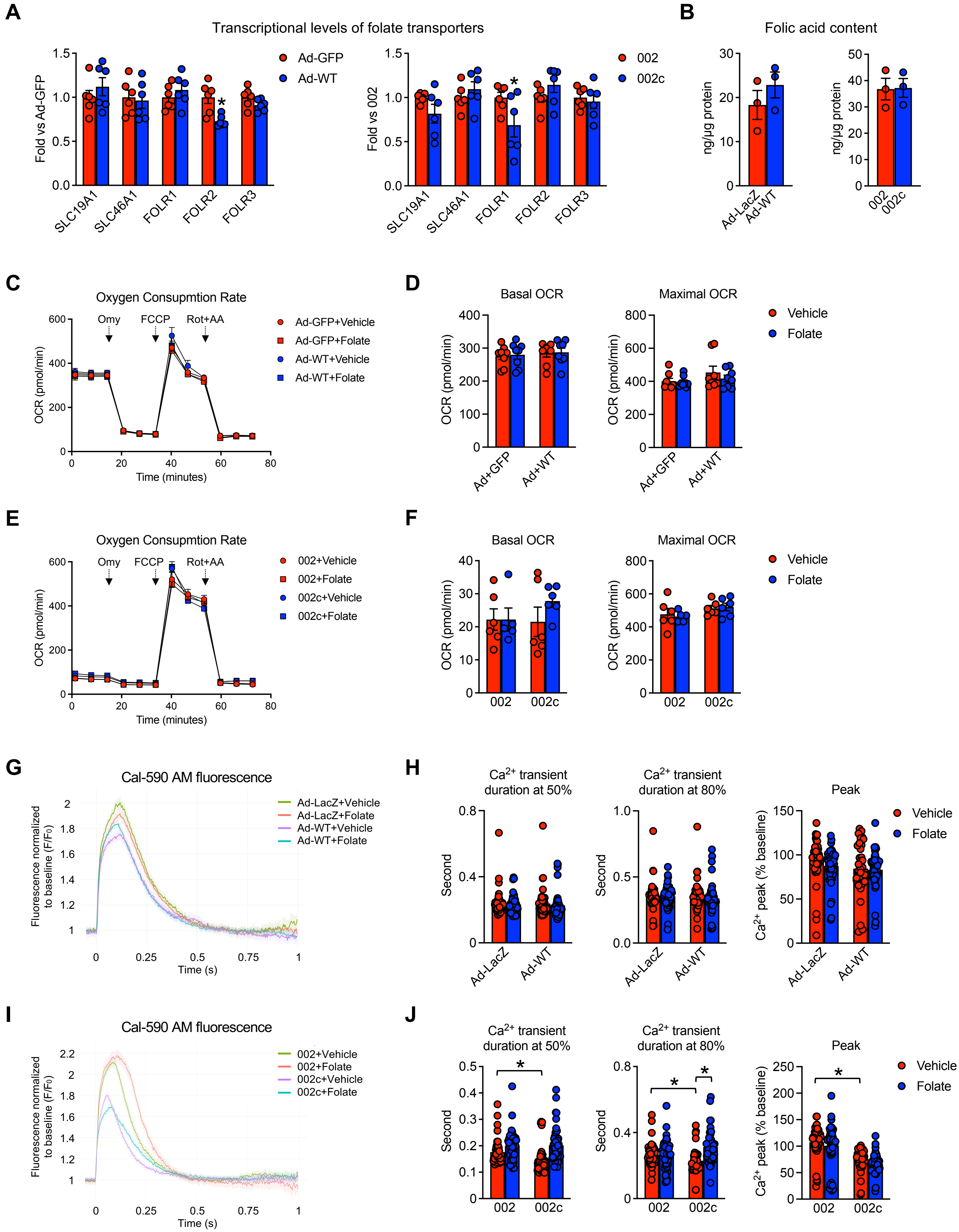

Supplemental Figure 6. The anti-arrhythmic effect of folate is unrelated to OXPHOS and calcium handling capacity in TDD iPSC-CMs. (**A**) Transcriptional level of five plasma folate transporters in TAN016+Ad-GFP/+Ad-WT (left panel) and TAN002/TAN00c (right panel) iPSC-CMs. Quantification was performed by RT-PCR (n=6). *PPIB* was used as internal control. *p<0.05 vs Ad-GFP (left panel) or 002 (right panel). (**B**) Baseline folic acid level in TAN016+Ad-GFP/+Ad-WT (left panel) and TAN002/TAN00c (left panel) iPSC-CMs (n=3). The folic acid level was normalized to total protein level. (**C and D**) Oxygen consumption rate (OCR) measurement for TAN016+Ad-GFP/+Ad-WT iPSC-CMs with vehicle or folate (n=8). Arrows in C indicate drug injection of 3 μM oligomycin (Omy), 0.5 μM FCCP, 5 μM rotenone (Rot) and 5 μM antimycin A (AA). Quantification of basal OCR (left panel) and maximal OCR (right panel) is shown in D. (**E and F**) OCR measurement for TAN002 and TAN002c iPSC-CMs with vehicle or folate (n=5-6). Arrows in C indicate drug injection. Basal OCR (left panel) and maximal OCR (right panel) are shown in F. (**G and H**) Calcium transient analysis for TAN016+Ad-LacZ/+Ad-WT iPSC-CMs with vehicle or folate. Panel G shows the representative fluorescence recording tracings at 1Hz pacing. Panel H shows the quantification (n=46-48). (**I and J**) Calcium transient analysis for TAN002 and TAN002c with vehicle or folate. Panel I shows the representative fluorescence recording tracings at 1Hz pacing. Panel J shows the quantification (n=46-51). *: p<0.05, two-way ANOVA corrected by Sidak method (α=0.05). Data are mean + SEM. Error bars smaller than the symbol size are not shown.

Supplemental Table 1. List of qPCR primers

Target Gene	Forward primer Sequence (5' to 3')	Reverse primer Sequence (5' to 3')	Roche Probe Number
MYL2	GCAGGCGGAGAGGTTTTC	AGTTGCCAGTCACGTCAGG	63
MYH7	AGGAGCTCACCTACCAGACG	TGCAGCTTGTCTACCAGGTC	62
MYH6	CGACAAGATTGAGGACATGG	CGCTCCTTGAGGTTGAAAAG	4
SCN5A	GGCTCGACTTCCTCATCGTA	TCCGCAGTGACTTGATGG	64
KCNIP2	TGTACCGGGGCTTCAAGA	AAGGCATTGAAGAGAAAAGTGG	67
RYR2	GCGGATCCAGTAAAACACTTG	TCCCCATGTGGATAGTGTCAT	63
ATP2A2	GAAGAGAAGAAATGCACCTTGAG	CCAACGAAGGTCAGATTGGT	53
PPIB	GATGTCCAGGCCGATGCTG	CAAGCATGTGGTGTTTGGCA	10
KCNJ2	TGTCACGGATGAATGCCCAA	CAAACACAGCTTGCCGTCTC	-
SLC19A1	CTTTGCCACCATCGTCAAGACC	GGACAGGATCAGGAAGTACACG	-
SLC46A1	ATGCAGCTTTCTGCTTTGGT	GGAGCCACATAGAGCTGGAC	-
FOLR1	AGGTGCCATCTCTCCACAGT	GAGGACAAGTTGCATGAGCA	-
FOLR2	CTGGCTCCTTGGCTGAGTTC	GCCCAGCCTGGTTATCCA	-
FOLR3	CCTGGATCCGGCAGGTC	CACAGGGGCACGTTCAGA	-

Supplemental Video 1. TAN016 iPS-CMs after differentiation before replating

Supplemental Video 2. TAN016 iPSC-CMs after replating and infected with Ad-GFP

Supplemental Video 3. TAN016 iPSC-CMs after replating and infected with Ad-WT

Supplemental Video 4. TAN002 iPS-CMs after differentiation before replating

Supplemental Video 5. TAN002 iPS-CMs after replating

Supplemental Video 6. TAN002corr iPS-CMs after differentiation before replating

Supplemental Video 7. TAN002corr iPS-CMs after replating
