# Supplementary material for: Folate as a potential treatment for lethal ventricular arrhythmias in TANGO2-deficiency disorder
Source: JCI Insight. 2024 Jun 10;9(11):e171005. doi: 10.1172/jci.insight.171005 (PMC11382877; doi:10.1172/jci.insight.171005)

Full unedited gel for Figure 2B

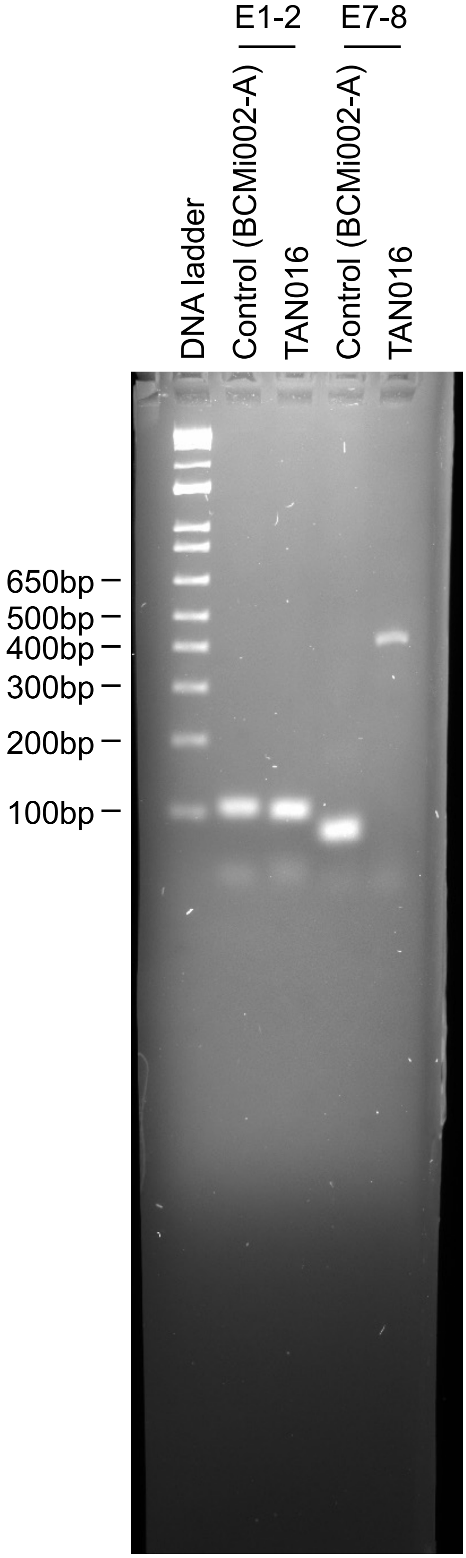

Full unedited gel for Figure 2D left panel

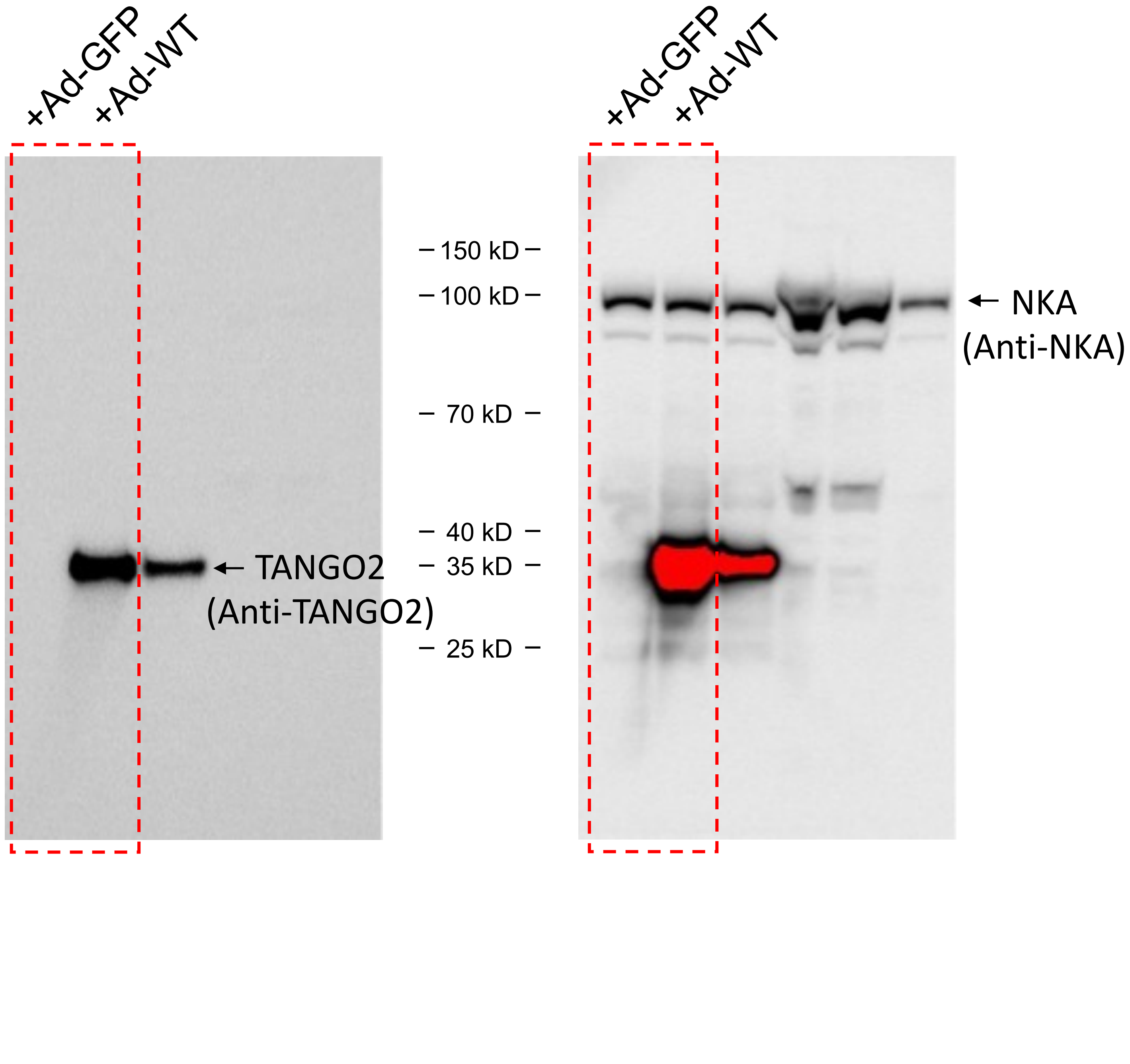

Full unedited gel for Figure 2D right panel

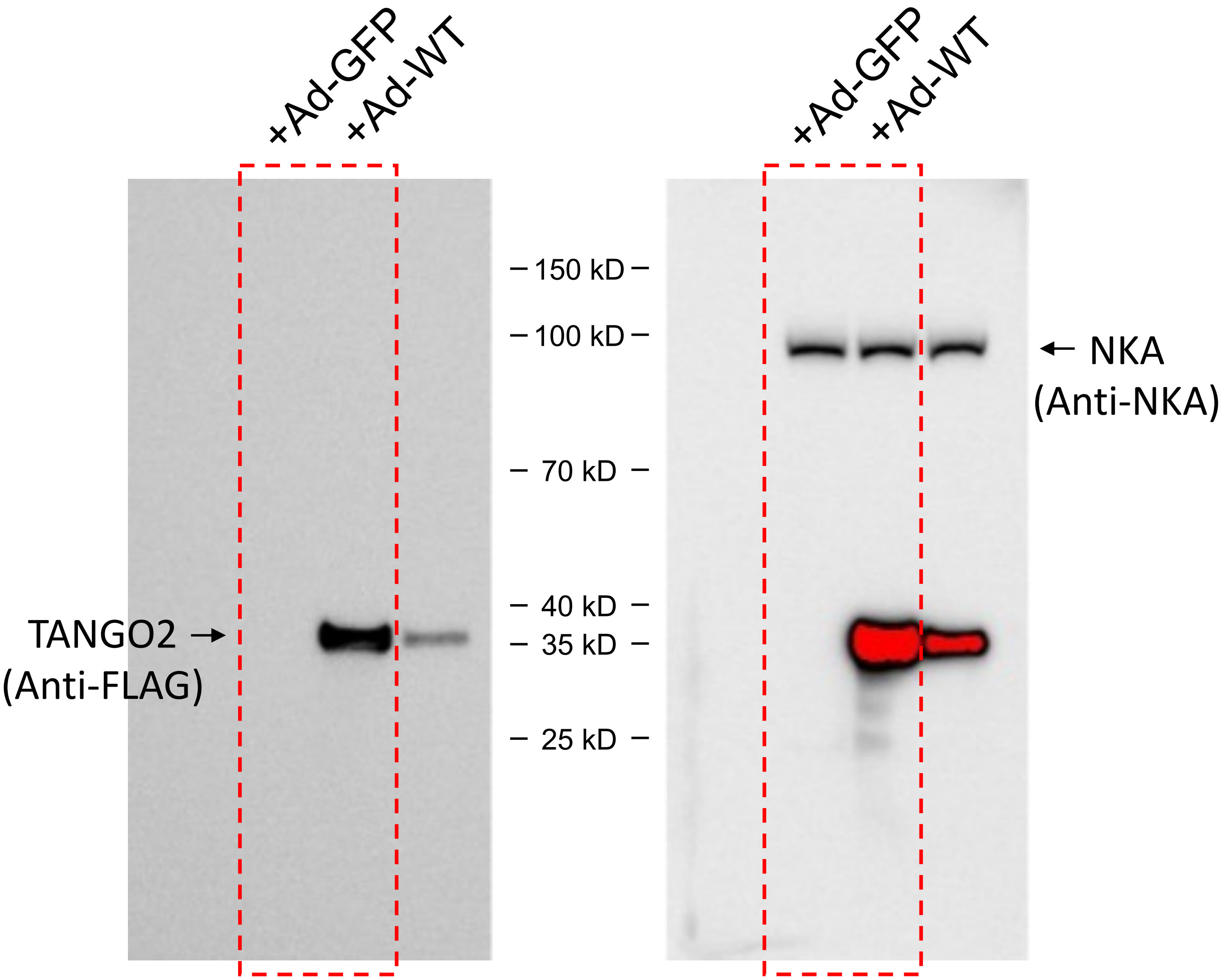

Full unedited gel for Figure 2F

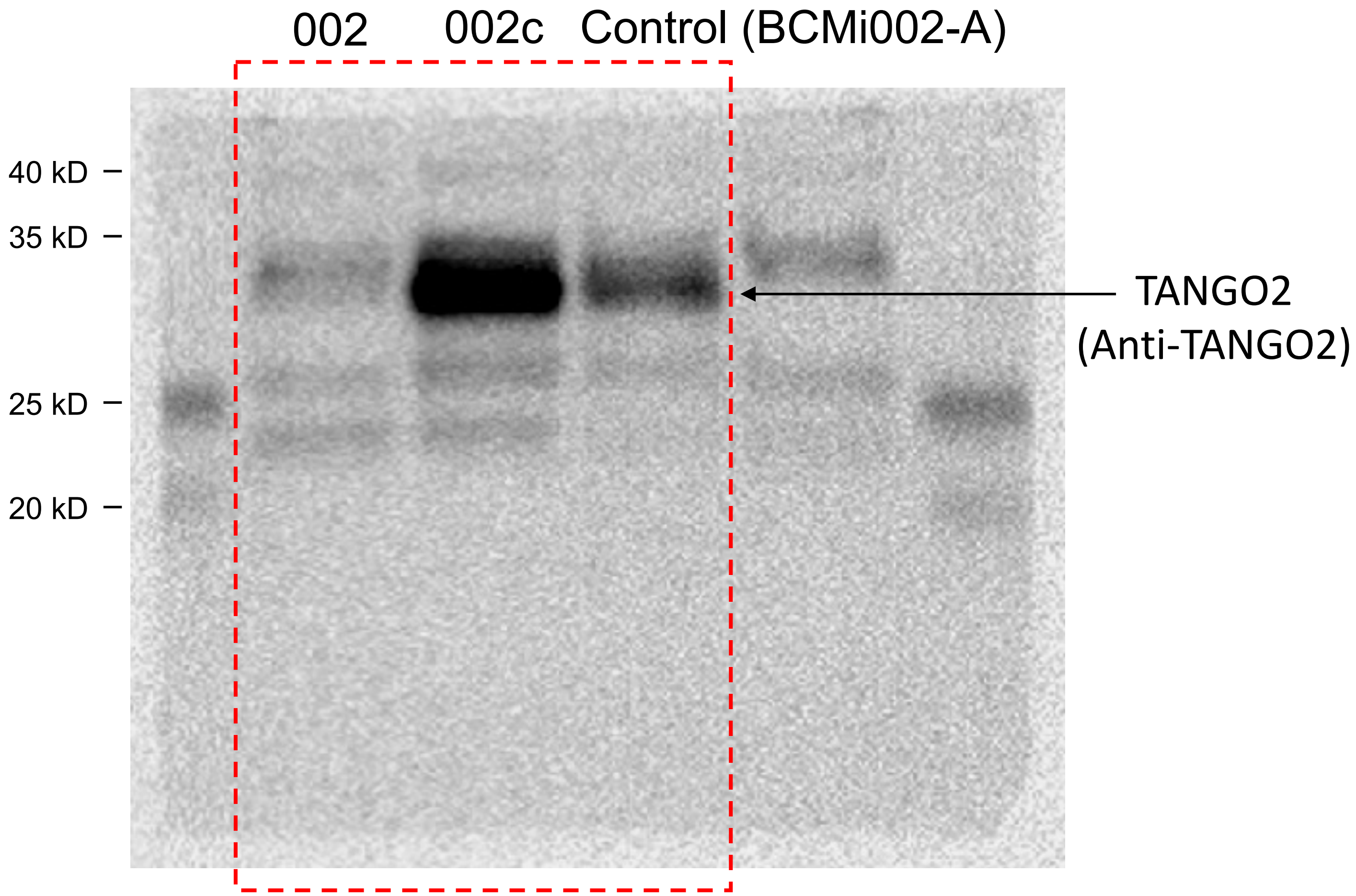

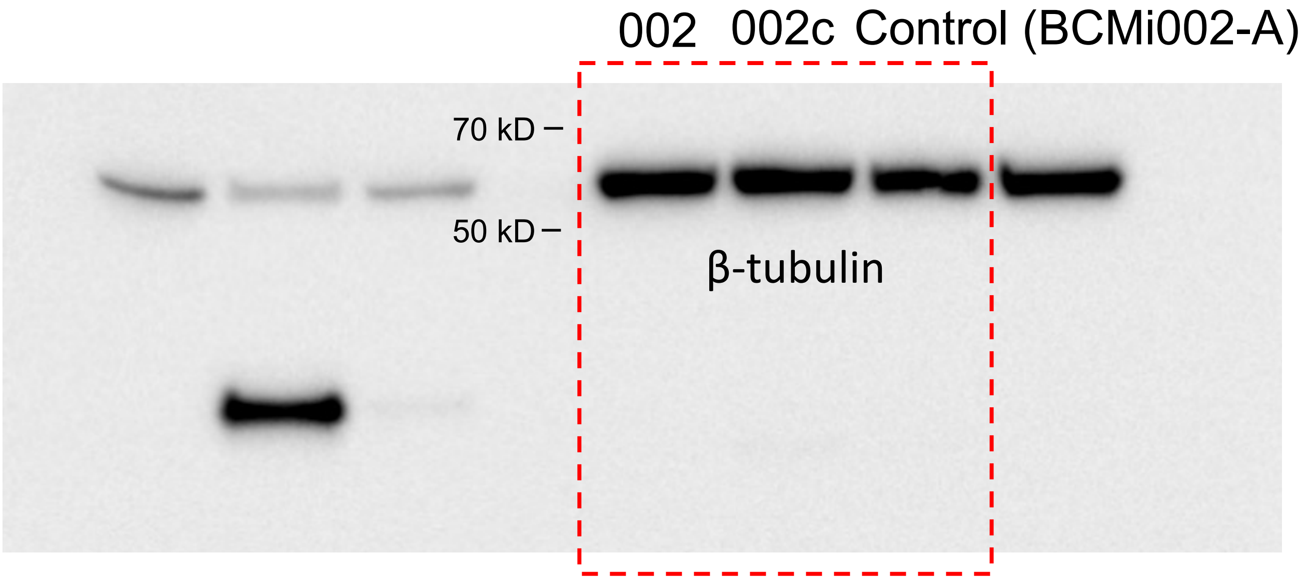

Supplement: Unedited blot and gel images [file jciinsight-9-171005-s052.pdf]
